# Evasion of cGAS and TRIM5 defines pandemic HIV

**DOI:** 10.1038/s41564-022-01247-0

**Published:** 2022-10-26

**Authors:** Lorena Zuliani-Alvarez, Morten L. Govasli, Jane Rasaiyaah, Chris Monit, Stephen O. Perry, Rebecca P. Sumner, Simon McAlpine-Scott, Claire Dickson, K. M. Rifat Faysal, Laura Hilditch, Richard J. Miles, Frederic Bibollet-Ruche, Beatrice H. Hahn, Till Boecking, Nikos Pinotsis, Leo C. James, David A. Jacques, Greg J. Towers

**Affiliations:** 1 Division of Infection and Immunity, UCL, London, UK; 2 EMBL Australia Node in Single Molecule Science, School of Medical Sciences, UNSW Sydney, Sydney, New South Wales Australia; 3 Department of Medicine, University of Pennsylvania, Philadelphia, PA USA; 4 Department of Microbiology, University of Pennsylvania, Philadelphia, PA USA; 5 Institute of Structural and Molecular Biology, Birkbeck College, London, UK; 6 MRC Laboratory of Molecular Biology, Cambridge, UK; 7 Present Address: Quantitative Biosciences Institute (QBI), University of California San Francisco, San Francisco, CA USA; 8 Present Address: Carnall Farrar, London, UK; 9 Present Address: Quell Therapeutics Ltd, Translation & Innovation Hub, London, UK; 10 Present Address: Nucleus Global, London, UK

**Keywords:** HIV infections, Viral immune evasion

## Abstract

Of the 13 known independent zoonoses of simian immunodeficiency viruses to humans, only one, leading to human immunodeficiency virus (HIV) type 1(M) has become pandemic, causing over 80 million human infections. To understand the specific features associated with pandemic human-to-human HIV spread, we compared replication of HIV-1(M) with non-pandemic HIV-(O) and HIV-2 strains in myeloid cell models. We found that non-pandemic HIV lineages replicate less well than HIV-1(M) owing to activation of cGAS and TRIM5-mediated antiviral responses. We applied phylogenetic and X-ray crystallography structural analyses to identify differences between pandemic and non-pandemic HIV capsids. We found that genetic reversal of two specific amino acid adaptations in HIV-1(M) enables activation of TRIM5, cGAS and innate immune responses. We propose a model in which the parental lineage of pandemic HIV-1(M) evolved a capsid that prevents cGAS and TRIM5 triggering, thereby allowing silent replication in myeloid cells. We hypothesize that this capsid adaptation promotes human-to-human spread through avoidance of innate immune response activation.

## Abstract (web summary)

Innate immune evasion is key to evolution of the pandemic lineage of HIV.

## Main

HIV-1 is derived from zoonotic infections from chimpanzees and gorillas^1,2^ and HIV-2 from sooty Mangabeys^3^. Most zoonoses do not lead to pandemic levels of human-to-human transmission, with pandemics being defined by the WHO (World Health Organization) according to both numbers of infections and global proliferation. Indeed, the numbers of people infected with non-pandemic HIV-1 are low. For example, non-pandemic HIV-1(P) has only been detected in 2 patients, HIV-1(N) in fewer than 20 patients and HIV-1(O) in fewer than 100,000 patients, well below the 80,000,000 infections caused by pandemic HIV-1(M). Similarly, the two zoonoses from sooty mangabeys leading to HIV-2 A and B have caused fewer than 2,000,000 infections, with numbers in decline^4,5^. HIV-2 C-I have only been detected in single patients and HIV-2(F) in only 2 patients from the same geographical region^6–9^. Thus, pandemics are rare and the specific adaptations underlying HIV-1(M) pandemicity are poorly understood. Previous phylogenetic studies have suggested chance events^10^, while molecular studies have identified HIV-1(M) Vpu as a uniquely effective human tetherin antagonist^11,12^, and non-POU domain-containing octamer-binding protein (NONO) as targeting and inhibiting HIV-2 capsids but not HIV-1(M)^13^.

The HIV core, built of capsid protein, accommodates and regulates viral DNA synthesis^14,15^, hence we hypothesized that capsid adaptations might favour pandemicity by preventing exposure of viral DNA to the cytoplasmic innate immune sensor cGAS^16–21^. HIV capsid is known to function as a pathogen-associated molecular pattern (PAMP) and may also be differentially recognized by the restriction factor TRIM5 (ref. ^22^). HIV-1(M) has been reported to be insensitive to human TRIM5 because cyclophilin A (CypA) shields incoming HIV-1(M) cores^23,24^. However, simian TRIM5 variants can form a restrictive hexameric cage around HIV-1(M) cores, which inhibits viral uncoating and nuclear entry^25–27^. Coordination of TRIM5 trimers at cage vertices facilitates TRIM5-mediated K63 linked ubiquitin (Ub) chain synthesis and activation of AP-1 and NF-kB transcription factors^22,28,29^. cGAS and TRIM5 therefore activate pro-inflammatory signalling, which suppresses viral replication^16,22^. We hypothesized that pandemic HIV-1(M) might be particularly effective in avoiding innate immune sensing because otherwise, early inflammatory responses should limit transmission^30^. We set out to test this hypothesis and report our findings here.

## Results

### Non-pandemic HIV isolates activate innate immunity

**Fig. 1:**
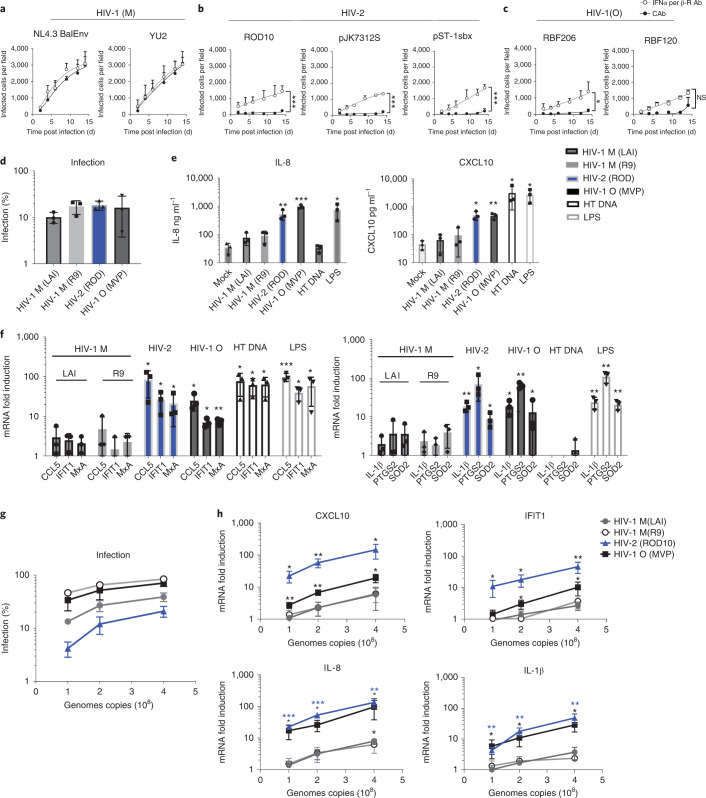
HIV activation of innate immune responses in macrophages. **a**–**c**, Replication of HIV-1(M) (**a**), HIV-2 (**b**) or HIV-1(O) (**c**) isolates in human MDM in the presence of interferon α/β receptor (IFNα/β-R) or control antibody (CAb). Two-way ANOVA vs CAb, ROD10 *P* = 0.0001, pSTbx *P* = 0.0001, ps7312s *P* = 0,0001, RBF206 *P* = 0.033. **d**, Single-round infection of MDM with equal genome copies of VSV-G-pseudotyped HIV-1(M), HIV-2 and HIV-1(O)-GFP measured 48 h post infection. **e**, Secreted IL-8 and CXCL10 from infections in **d** measured by ELISA 48 h post infection. **f**, GAPDH-normalized mRNA levels in infections from **d** expressed as fold induction over untreated MDM 24 h post infection or after HT-DNA transfection (1 ug ml^−1^) or LPS stimulation (100 ng ml^−1^). **g**, Infection of THP-1 cells with equal genome copies of VSV-G-pseudotyped HIV-1(M), HIV-2 and HIV-1(O)-GFP measured 48 h post infection. **h**, GAPDH-normalized mRNA levels from infections in **g** expressed as fold induction over untreated THP-1 cells 24 h post infection. Mean ± s.d., *n* = 3 donors (**a**–**e**) or independent experiments (**f**–**h**). Two-tailed unpaired *t*-test vs untreated MDM (**d**–**f**), paired *t*-test vs untreated THP-1 cells (**g**,**h**). *P < 0.05, **P < 0.01, ***P < 0.001. NS, not significant. Source data

**Extended Data Fig. 1.**
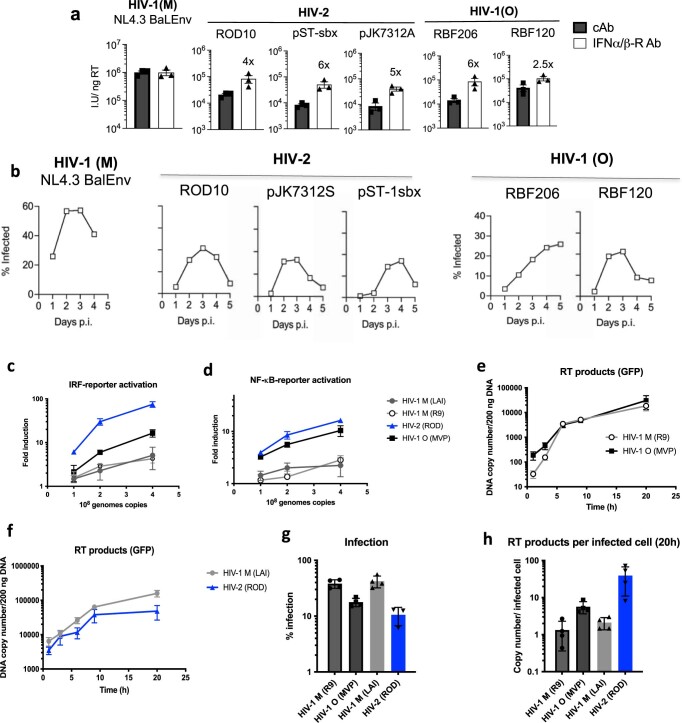
**a. Infection of MDM with HIV, measured at 48** **h by counting Gag positive cells, in the presence of anti-interferon α/β receptor (IFNα/β-R), or control, antibody (cAb).** **b**. Replication of HIV-1(M), HIV-2 or HIV-1(O) isolates in permissive GHOST cells measuring induced GFP expression by flow. Representative experiment of 2 independent replicates. **c, d**. Activation of (c) IRF-luciferase reporter or (d) NF-kB secreted alkaline phosphatase reporter 48 h after infection by equal genome copies of VSV-G-pseudotyped HIV-1(M), HIV-2 or HIV-1(O) -GFP. **e, f**. Measurement of VSV-G pseudotyped HIV-1(M), HIV-1(O) and HIV-2 DNA synthesis (GFP primers) during a 20 h time course in THP-1 cells. **g**. Infection measured at 48 hours in wells parallel to (e) by flow. **h**. Viral DNA (GFP) copy number at 20 h post-infection per infected cell using data from (e-f). Mean + /− SD, N = 3 donors (MDM) or independent experiments (THP-1 c,d). N = 4 independent experiments ThP1 e-h. Source data

Pandemic HIV-1(M) isolates infect macrophages efficiently in vitro, in mouse models and in vivo^31–34^. Conversely, HIV-2 fails to replicate in macrophages and dendritic cells, partly because increased viral DNA synthesis due to Vpx-mediated degradation of SAMHD1 is sensed by cGAS, followed by innate immune activation^17,35,36^. We found that neither HIV-2 (has Vpx) nor HIV-1(O) (lacks Vpx) can replicate in primary human monocyte-derived macrophages (MDM) unless type-I interferon (IFN) signalling is suppressed using IFN receptor (IFNAR1) antibody (Ab) (Fig. 1a–c). However, HIV-1(M) replicated well in MDM, and IFNAR1-Ab had no effect on replication (Fig. 1a)^31^. IFNAR1-Ab also increased single-round MDM infection by HIV-2 and HIV-1(O), but not HIV-1(M) (Extended Data Fig. 1a). All viruses replicated efficiently in permissive GHOST^37^ cells, demonstrating fitness (Extended Data Fig. 1b). Concordantly, infection of MDM with equal genome copies (measured by quantitative PCR with reverse transcription (RT-qPCR)) of vesicular stomatitis virus-G (VSV-G)-pseudotyped HIV-2 and HIV-1(O) GFP-encoding vectors, hereafter referred to as HIV-GFP, induced expression of interferon-stimulated genes (ISGs) (CCL5, IFIT1, MxA, CXCL10) and pro-inflammatory genes (IL-8, IL-1β, PTGS2 and SOD2), with IL-8 and CXCL10 secretion evidenced by ELISA (Fig. 1d–f). Despite similar infectivity, equal genome copies of HIV-1(M) induced less ISG and cytokine expression, consistent with interferon-independent replication in MDM (Fig. 1a,d–f). DNA transfection and lipopolysaccharide (LPS) treatment acted as positive controls for PAMP (Fig. 1e,f).

The striking difference in innate immune activation between pandemic and non-pandemic viruses was reproduced upon infection in the myeloid cell line model, undifferentiated THP-1 cells (Fig. 1g,h)^38^. Using VSV-G-pseudotyped GFP-encoding vectors and equalizing the amount of virus by genome copies (RT-qPCR), we found that HIV-2 and HIV-1(O) induced dose-dependent ISG and cytokine expression to higher levels than HIV-1(M), even though HIV-1(M) infection levels were higher when using equivalent amounts of viral DNA (Fig. 1g,h). Additionally, THP-1 cells harbouring an ISG54 minimal promoter regulated by five IFN-stimulated response elements (IRF), or an NF-kB-sensitive reporter, were activated to higher levels by HIV-2 and HIV-1(O) compared with HIV-1(M) infection (Extended Data Fig. 1c,d). Importantly, measurement of viral DNA synthesis after equalizing genome input revealed higher levels of infection but similar levels of reverse transcripts for HIV-1(M), also ruling out increased DNA synthesis by HIV-2/HIV-1(O) as the reason for increased innate activation (Extended Data Fig. 1e–g). Indeed, HIV-1(M) is more efficient in infecting THP-1 cells per reverse transcript (Extended Data Fig. 1e–g), suggesting that non-infectious HIV-2/HIV-1(O) DNA might activate innate immune responses. Plotting reverse transcriptase (RT) products per infected cell also revealed infection efficiency differences: HIV-1(M) uniquely synthesizes close to one molecule of DNA per infectious unit in these cells (Extended Data Fig. 1h). Crucially, VSV-G-pseudotyped near-full-length HIV-1(M) clone LAI deleted for Envelope (pLAI∆Env) ∆Env^39^ (labelled LAI) and minimal HIV-1(M) R9-based GFP-encoding vector (p8.91 + CSGW^40,41^, labelled R9) gave similar low-level stimulation in MDM and THP-1 (Fig. 1e–h).

### cGAS and TRIM5 detect non-pandemic HIV isolates

**Fig. 2:**
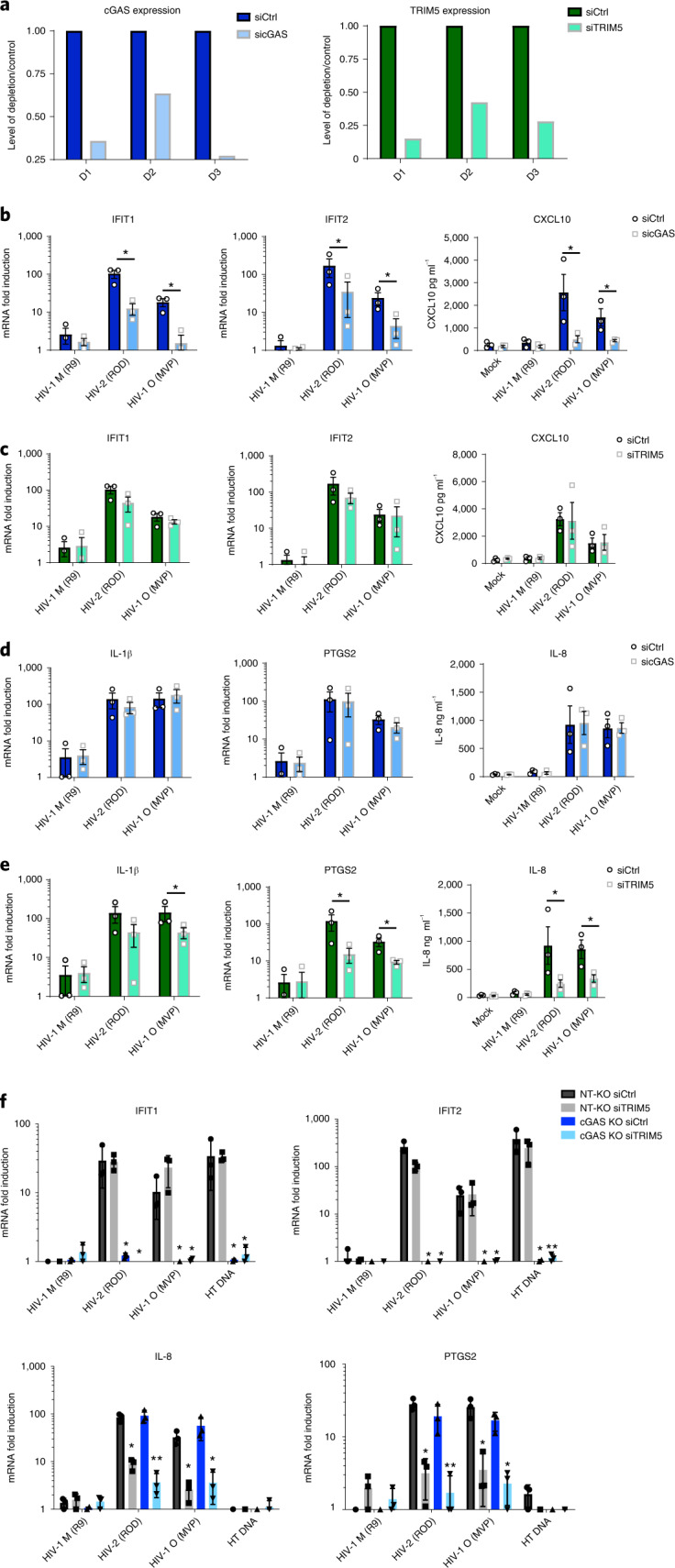
cGAS and TRIM5 detect non-pandemic HIV. **a**, Example of GAPDH-normalized cGAS mRNA levels in MDM representing 3 independent donors transfected with cGAS-targeting siRNA (sicGAS), TRIM5-targeting siRNA (siTRIM5) or non-targeting control (siCtrl). **b**–**e**, GAPDH-normalized mRNA levels (left and middle), or secreted cytokine levels (right) (ELISA) (CXCL10, IL-8), expressed as fold induction over uninfected (mock) samples 24 h post infection (mRNA) or 48 h post infection (cytokine) from cells in **a**. **f**, GAPDH-normalized mRNA levels, expressed as fold induction over uninfected samples, in non-targeting CRISPR-treated cells (NT-KO) transfected with control siRNA (siCtrl) (NT-KO siCtrl), NT-KO siTRIM5, cGAS KO siCtrl or cGAS KO siTRIM5 THP-1 cells 24 h post infection or after HT-DNA (1 ug ml^−1^) transfection. Mean ± s.d., *n* = 3 independent experiments and donors. Two-tailed unpaired *t*-test vs untreated MDM (**b**–**e**), paired *t*-test vs THP-1 Ctrl vector (**f**). *P < 0.05, **P < 0.01. Source data

We hypothesized that innate immune activation by non-pandemic viruses reflected greater sensitivity to innate immune sensors. Indeed, RNAinterference (RNAi)-mediated depletion of well-characterized sensors cGAS and TRIM5 (Fig. 2a) reduced induction of cGAS- and TRIM5-specific genes by HIV-2 and HIV-1(O) in MDM. Specifically, induction of IFIT1, IFIT2 and CXCL10 by HIV-2 and HIV-1(O) was reduced by cGAS (Fig. 2b) but not TRIM5 depletion (Fig. 2c). Conversely, induction of IL-1β, PTGS2 and IL-8 were reduced by TRIM5 (Fig. 2e) but not cGAS depletion (Fig. 2d). We also note that PTGS2, IL-1β and IL-8 were also not induced by transfecting DNA, consistent with cGAS insensitivity (Fig. 1e,f). As before, pandemic HIV-1(M) induced gene expression relatively poorly (Fig. 2b–e).

**Extended Data Fig. 2:**
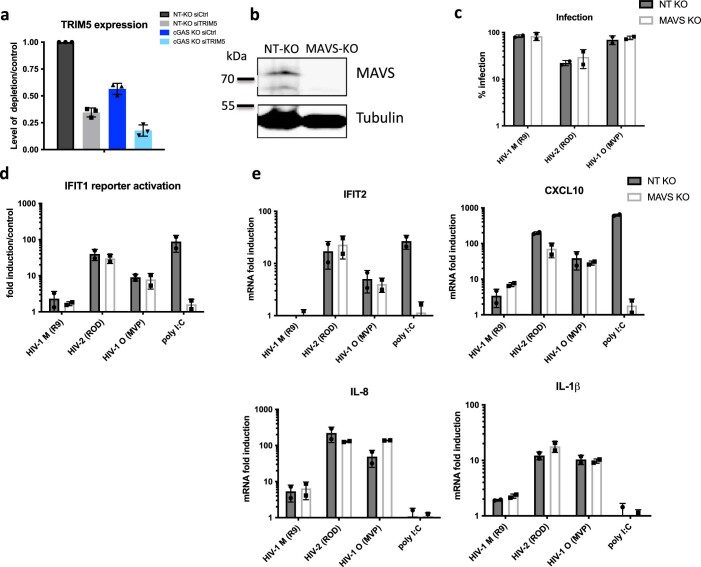
**a. GAPDH-normalised TRIM5 mRNA levels, measured after transfection of THP-1 treated with non-targeting CRISPR (NT-KO) or cGAS CRISPR (cGAS-KO) and transfected with TRIM5 targeting siRNA (siTRIM5) or non-targeting control siRNA (siCtrl).** N = 3 independent experiments. **b**. Anti-MAVS or tubulin western blot of non-targeting KO cells (NT-KO) or MAVS KO cells. **c**. % infection of HIV-1(M), HIV-2 and HIV-1(O) in NT-KO or MAVS KO cells. **d**. IFIT1 reporter activation after viral infection normalised against mock infected. **e**. GAPDH-normalised mRNA levels expressed as fold induction over mock-treated non-targeting KO control (NT-KO) or MAVS KO THP-1 cells 24 h post-infection or after poly I:C transfection (500 ng/mL). Mean + /− SD, n = 2 independent experiments. Source data

Sensor-specific gene induction by HIV-2 and HIV-1(O) was confirmed in commercial (Invivogen) THP-1 knockout (KO) lines where cGAS KO abrogated IFIT1 and IFIT2 induction, but did not particularly impact induction of TRIM5-sensitive IL-8 and PTGS2 (Fig. 2f, dark blue bars). By contrast, small-interference (siRNA)-mediated depletion of TRIM5 in THP-1 (Extended Data Fig. 2a) reduced TRIM5-sensitive IL-8 and PTGS2 induction, but had little effect on cGAS-sensitive IFIT1 and IFIT2 (Fig. 2f, grey bars). Importantly, depleting TRIM5 with siRNA in the cGAS KO line (Extended Data Fig. 2a) reduced all inflammatory gene expression (Fig. 2f, light blue bars). Mitochondrial anti-viral signalling protein (MAVS) KO had no impact on innate immune activation by HIV-2/HIV-1(O) (Extended Data Fig. 2b–e). These data suggest that cGAS and TRIM5 independently contribute to the inflammatory innate immune response to non-pandemic lentivirus infection in macrophages.

### Nuclease TREX1 suppresses innate sensing of non-pandemic HIV

**Extended Data Fig. 3:**
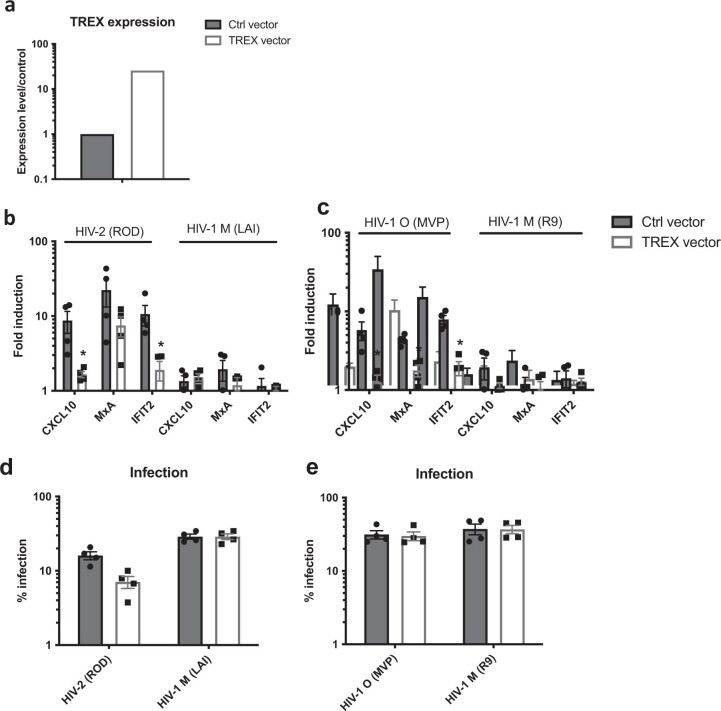
**a. A representative example of GAPDH-normalised TREX1 mRNA levels measured after transduction of THP-1 cells with empty (Ctrl) MLV vector or TREX1 expression vector.** **b, c**. GAPDH-normalised ISG mRNA levels, expressed as fold induction over uninfected, in control vector (Ctrl) or TREX1-expressing THP-1 cells with HIV-2 and HIV-1(M) or HIV-1(O) with **d, e**. infection levels in parallel wells measured 48 hpi. Mean + /− SD, n = 3 independent experiments. b,d two-tailed paired t-test vs THP-1 Ctrl vector. *p < 0.05. Source data

Depletion of cytoplasmic nuclease TREX1 has been shown to cause HIV-1(M) DNA to activate cGAS^42,43^. This suggests that cGAS detects DNA released from prematurely uncoating viral particles if not degraded by TREX1. We therefore tested whether TREX1 over-expression in THP-1 could degrade the cGAS-activating viral DNA from non-pandemic viruses and suppress sensing. In fact, 20-fold TREX1 mRNA over-expression (Extended Data Fig. 3a) reduced ISG induction by both HIV-2 and HIV-1(O), consistent with their DNA activating innate immune responses (Extended Data Fig. 3b,c). Unexpectedly, TREX1 over-expression also reduced HIV-2, but not HIV-1(O) infectivity (Extended Data Fig. 3d,e), indicating that HIV-2 capsids cannot effectively shield viral DNA from high TREX1 levels. This suggests that TREX1-sensitive HIV-2 cores are infectious and that HIV-2 and HIV-1(O) differ in their uncoating strategies, perhaps regarding timing and location, leading to different mechanisms of TREX1 sensitivity.

### Genome-free HIV activates TRIM5 and an antiviral state

**Fig. 3:**
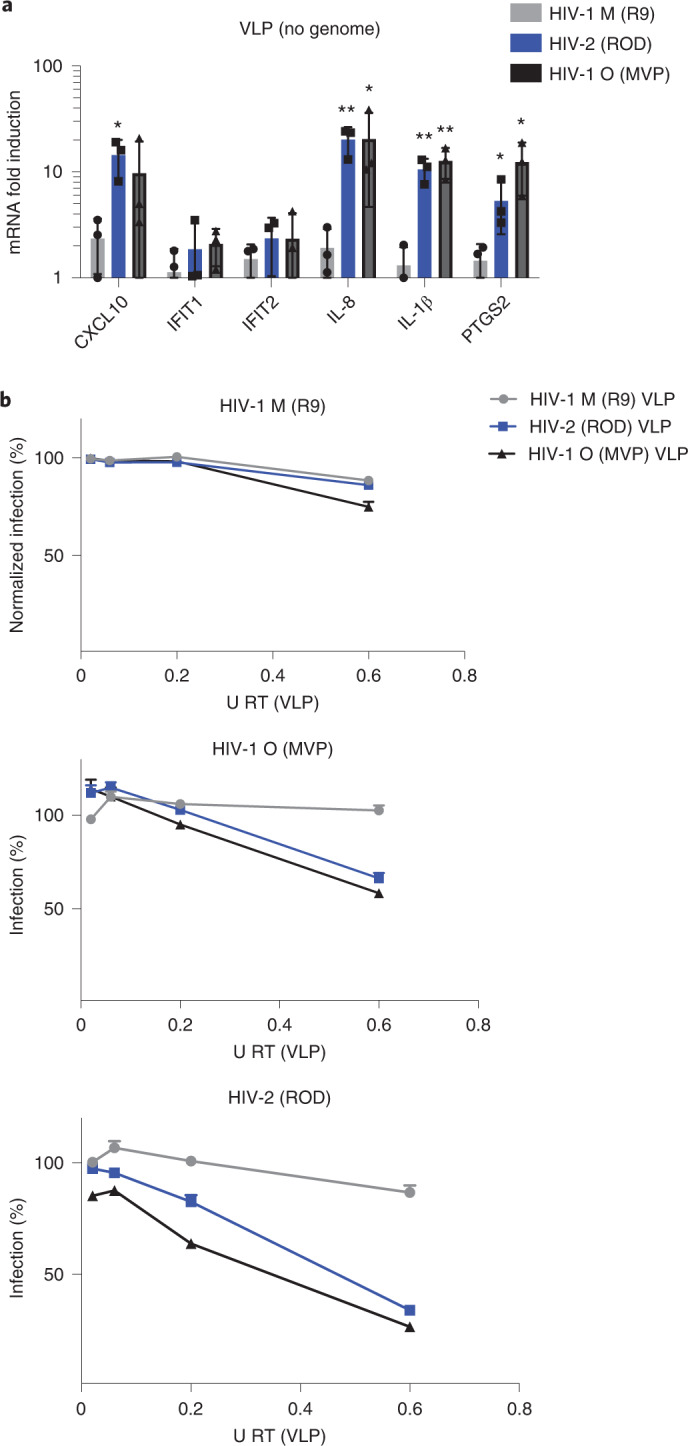
Genome-free HIV activates TRIM5, but not cGAS, to induce an antiviral state. **a**, GAPDH-normalized mRNA levels expressed as fold induction over uninfected THP-1 cells 24 h post viral-like particles (VLP) treatment. **b**, HIV-1(M), HIV-2 and HIV-1(O) infection levels normalized to levels of infection without previous exposure to VLP. Mean ± s.d., *n* = 3 independent experiments. Two-tailed paired *t*-test vs untreated THP-1 (**a**). *P < 0.05, **P < 0.01. VSV-G-pseudotyped VLP were made without genome. Source data

**Extended Data Fig. 4:**
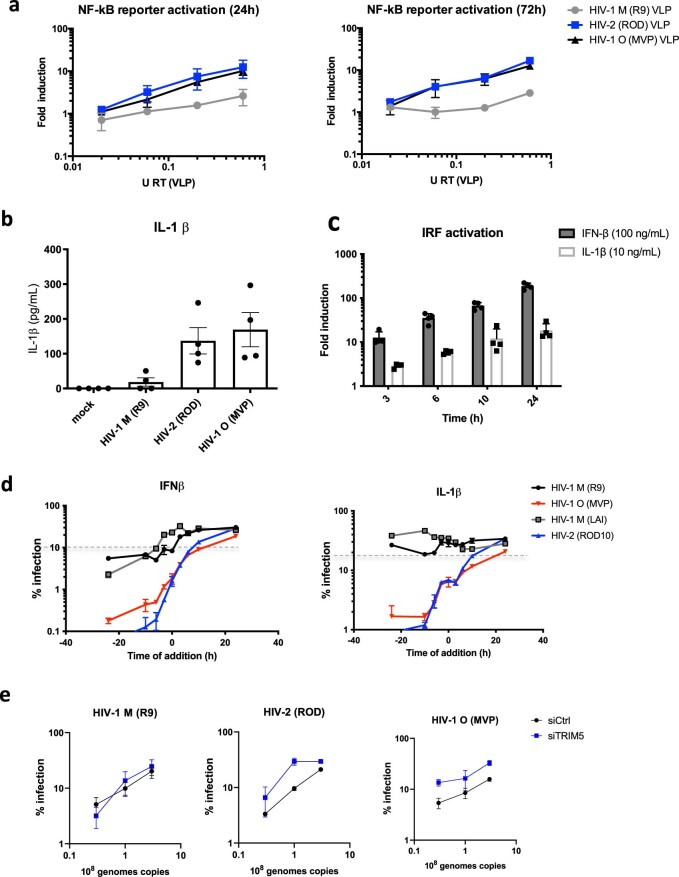
**a. NF-kB - secreted alkaline phosphatase reporter 24 or 72** **hours after treatment with increasing doses of viral-like particles (VLP, made with packaging plasmid and VSV-G but no genome).** N = 2 independent experiments. **b**. Quantification of IL-1β in supernatants from MDM mock infected or infected with HIV-1(M), HIV-2 or HIV-1(O). N = 4 donors. **c**. IRF-reporter activation after interferon β (IFNβ) or IL-1β treatment of THP-1 cells. N = 4 independent experiments. **d**. % of infection in THP-1 cells after addition of IFNβ or IL-1β at different time points. Dotted line indicates the % of infection of untreated cells. N = 3 independent experiments **e**. Infection levels in MDM depleted of TRIM5 with equal genome copies of VSV-G pseudotyped HIV-1(M), HIV-2 or HIV-1(O) –GFP measured 48 h post-infection. N = 2 donors. Data shows mean + SD. Source data

To assess TRIM5’s contribution to gene induction, we used VSV-G-pseudotyped viral-like particles (VLP) produced without genomes. As expected, genome-free VLP induced TRIM5-sensitive IL-8, IL-1β and PTGS2, but not cGAS-sensitive IFIT1/IFIT2 in THP-1 (Fig. 3a). To test whether TRIM5 activation induced an antiviral state in THP-1, we activated TRIM5 using increasing doses of genome-free VLP and then infected the same cells 24 h later. We observed dose-dependent activation of TRIM5-sensitive NF-kB reporter by HIV-2 and HIV-1(O), but very little activation by HIV-1(M) VLPs (Extended Data Fig. 4a). Importantly, responses to non-pandemic VLP inhibited a second infection by HIV-2 or HIV-1(O)-GFP (Fig. 3b). Consistent with the failure of HIV-1(M) to activate gene expression, HIV-1(M) VLPs did not induce an antiviral state (Fig. 3b). Surprisingly, HIV-1(M) was also insensitive to the effects of previous exposure to HIV-2 or HIV-1(O) VLPs (Fig. 3b). Thus, TRIM5 activation induces the expression of antiviral genes, including secretion of IL-1β (Extended Data Fig. 4b) that can restrict non-pandemic HIV. Concordantly, pre-treatment of THP-1 with IL-1β or IFN-β reduced infection of all viruses but with pandemic HIV-1(M) being notably less sensitive, even when target cells were pre-treated (Extended Data Fig. 4c,d). Importantly, single-round infections with all viruses were much less sensitive to IL-1β and IFN treatment when added, for example, 6 h post infection (Extended Data Fig. 4d), consistent with a model in which single-round infections induce cytokines too late to inhibit that first round of infection. This is also evidenced by modest rescue of single-round infection (GFP expression) of HIV-2 and HIV-1(O) when TRIM5 is depleted in macrophages (Extended Data Fig. 4e). We propose that sensor activation initiates responses that are more potent against later rounds of infection, evidenced by greater inhibition of infection when cells are pre-treated with cytokine (Extended Data Fig. 4d).

### Lineage-associated adaptation of HIV capsid at position 50

**Fig. 4:**
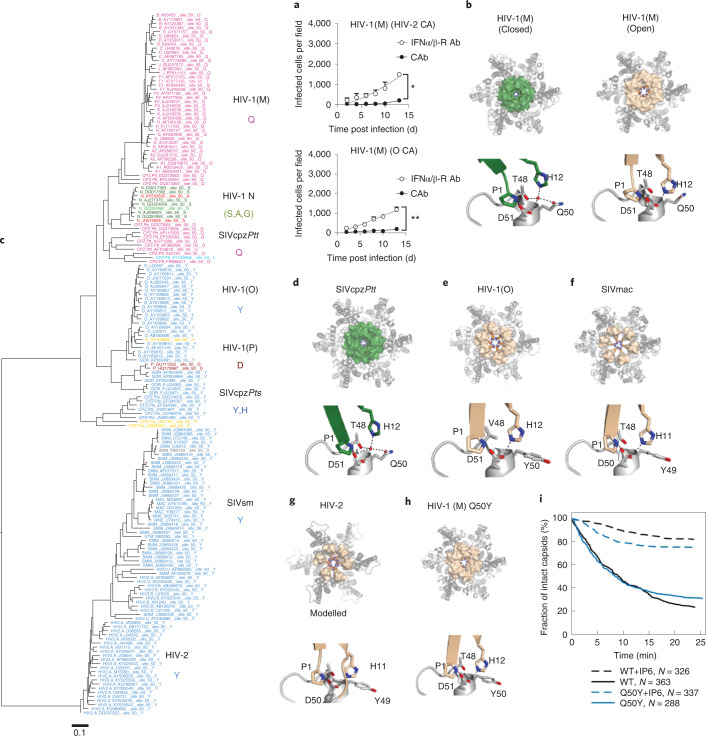
Pandemic-associated adaptation of HIV capsid at position 50. **a**, Replication of HIV-1(M) NL4.3 (BalEnv) bearing HIV-2 ROD10 (top) or (O) MVP5180 (bottom) capsid in MDM in the presence of IFNα/β-R or CAb. Data show mean + s.d. *n* = 3 donors. Two-way ANOVA vs CAb, HIV-1(HIV-2 CA) *P* = 0.02, HIV-1(O CA) *P* = 0.002. **b**, HIV-1(M) CA hexamer highlighting β-hairpin (BHP) position in a closed (green) (PDB ID:5HGN) or open conformation (wheat) (PDB ID:5HGL). Lower panel details residues in hinge region that close BHP by coordinating water and increasing distance between His12 and Asp51. **c**, Maximum-likelihood phylogenetic tree of primate lentiviral capsid genes coloured by chromaclade to illustrate the residues equivalent to CA Q50 in HIV-1(M). **d**, SIVcpz CA hexamer (PDB ID:7T15) with the BHP position in a closed (green) conformation. Lower panel details residues in hinge region that close BHP by coordinating water and increasing distance between His12 and Asp51. **e**,**f**, HIV-1(O) (PDB ID: 7T12) and SIVmac (PDB ID:7T14) hexamers with open BHP (wheat) and key Gln at position 50 substituted for Tyr (Y), preventing water coordination and channel closure. Lower panels show the BHP hinge region in detail. **g**, A modelled hexamer built from the HIV-2 N-terminal CA domain (PDB ID:2 × 82) is shown for comparison with HIV-1(M) and (O). The HIV-2 BHP (wheat) is open. Note that HIV-2 position 49 is Tyr (Y). **h**, Crystal structure of HIV-1(M) Q50Y (PDB ID:7T13) highlighting BHP position in an open conformation (wheat). R18 is shown at the centre of the hexamers. Lower panels show the BHP hinge region in detail. **i**, WT and CA Q50Y capsid survival curves obtained from TIRF in vitro uncoating experiments. In the absence of IP6, HIV-1 Q50Y capsids are metastable and disassemble spontaneously with similar half-life to WT. Most capsids are stabilized in the presence of 100 μM IP6. Survival curves were generated from single-virion uncoating traces (*N* = 326 for WT, 326 for WT + 100 μM IP6, 326 for Q50Y, 326 for Q50Y + 100 μM IP6) from one representative uncoating experiment (see Extended Data Fig. 5 for additional Q50Y data). Source data

**Extended Data Fig. 5:**
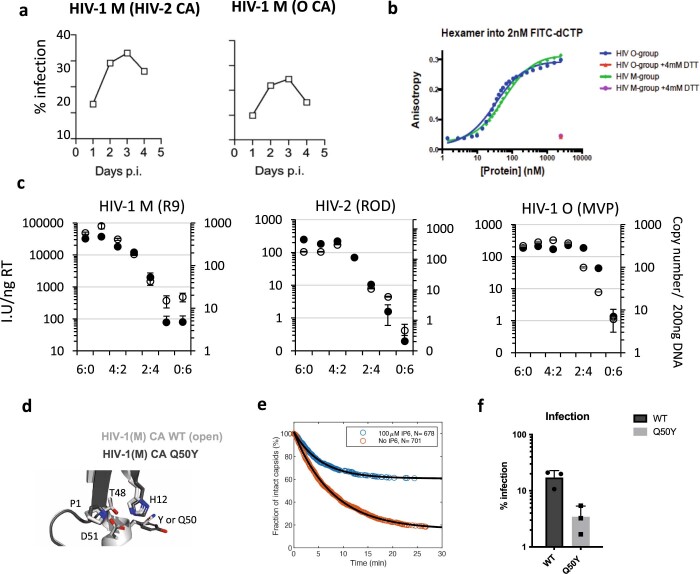
**a. Replication of HIV-1(M) NL4.3 (Bal Env) bearing HIV-2 ROD10 or HIV-1(O) MVP5180 Capsid in permissive GHOST cells (flow cytometry for induced GFP expression) representative of 2 independent experiments.** **b**. Binding of fluorescently labeled nucleotides to HIV-1(M) or HIV-1(O) recombinant CA hexamers in the presence or absence of DTT to reduce monomer cross links. **c**. Titres of HIV-1(M), HIV-2 or HIV-1(O) -GFP made by mixing WT and CA R18G bearing packing constructs (left axis, white circles) and DNA synthesis measured at 6 hours post infection (right axis, black circles). **d**. Amino acids in BHP hinge region influencing BHP position with overlay of HIV-1(O) (PDB ID:7T12) and open HIV-1(M) (PDB ID:5HGL) hexamers. **e**. Capsid survival curves for CA mutant Q50Y generated from pooled data from two (no IP6) or three (100 μM IP6) independent experiments showing IP-mediated capsid stabilisation. **f**. Single round infection of MDM with equal genome copies of VSV-G-pseudotyped HIV-1(M) (R9) CA Q50Y –GFP measured 48 h post-infection by flow. Mean + /− SD, n = 3 independent experiments and 3 donors for MDM. Source data

A key role for capsid in determining interferon induction in macrophages was identified by infection with HIV-1(M) chimeras bearing HIV-1(O) or HIV-2 capsid (CA), which could only replicate in MDM in the presence of IFNAR1-Ab (Fig. 4a), in common with wild type (WT) HIV-2/HIV-1(O)(Fig. 1a–c). Importantly, chimeric viruses replicated efficiently in GHOST cells, indicating fitness (Extended Data Fig. 5a). Previously reported X-ray structures of HIV-1(M) CA hexamers showed two distinct conformations with respect to a channel at the 6-fold symmetry axis. The beta hairpin structure (BHP) above the channel can assume ‘open’ (His12 forms a salt bridge with Asp51) or ‘closed positions’ (with a bridging water molecule between His12 and Asp51)(Fig. 4b)^14^. Inside the channel, 6 positively charged arginines (R18) are hypothesized to mediate electrostatic nucleotide recruitment to fuel encapsidated DNA synthesis^14,44^. HIV-1(O) and HIV-2 conserve arginines at the 6-fold symmetry axis^14^ and similar to HIV-1(M), purified recombinant HIV-1(O) hexamer binds deoxy-cytidine tri-phosphate (dCTP) with nanomolar dissociation constant (*K*_D_) (Extended Data Fig. 5b). Conservation of encapsidated DNA synthesis was also supported by chimeric viral particles with varying proportions of WT and Arg-to-Gly mutant CA protein. Increasing mutant CA proportionally reduced viral infectivity and DNA synthesis (Extended Data Fig. 5c). Indeed, DNA and infection profiles of WT/mutant mixtures were similar between HIV-1(O), HIV-2 and HIV-1(ref. ^14^), supporting conservation of the electrostatic channel, nucleotide recruitment and encapsidated DNA synthesis mechanisms.

Although capsids are broadly conserved, we investigated the potential for functional differences between pandemic and non-pandemic capsids using structural and evolutionary approaches. Chromaclade colours maximum-likelihood phylogenetic trees according to the amino acid present at each position in the alignment to highlight lineage-defining amino acids^45^. The coloured tree for CA position 50 (Fig. 4c) stood out because the amino acid at this position defines the pandemic lineage, with HIV-1(M) and its chimpanzee parent (SIVcpz*Ptt*) uniquely harbouring glutamine (Q). SIV from red-capped mangabeys (SIVrcm) (the parent providing capsid to SIVcpz*Ptt*^46^), SIVgor and HIV-1(O) all contain tyrosine (Y) at CA position 50, consistent with tyrosine being the ancestral state and Y50Q occurring in chimpanzees (Fig. 4c). Tyrosine is highly conserved in SIVsmm and maintained in HIV-2 (see PSSM Supplementary Table 1), supporting the notion that it is ancestral. Importantly, Y50Q requires two nucleotide changes (UAY to CAR) consistent with adaptive change. Strikingly, the two non-pandemic HIV-1(P) isolates both encode CA50D^6^, whereas HIV-1(N) isolates variously encode CA50S, (*n* = 6), CA50A (*n* = 2) or CA50G (*n* = 1), again requiring two nucleotide substitutions from the ancestral Q50. These examples suggest different evolutionary pathways for CA50 adaptation in the different lineages.

### Capsid X-ray structures reveal conformational adaptation

We solved the crystal structures of HIV-1(O) hexamers to 3 Å and found that unlike HIV-1(M), it exclusively adopted an ‘open’ channel conformation (Fig. 4e). Comparison of HIV-1(M) and HIV-1(O) hexamers explains the importance of position 50, which is conserved (Supplementary Table 1) and located in the BHP hinge region (compare Fig. 4b–e). In HIV-1(M), residue Q50 contributes to the tetrahedral hydrogen bonding network that promotes the ‘closed’ BHP position. This arrangement is also conserved in our parental SIVcpz*Ptt* capsid structure, with SIVcpz*Ptt* Q50 contributing to the ‘closed’ BHP position by coordinating a water molecule (Fig. 4d and Supplementary Table 1). However, in HIV-1(O), Y50 has been retained from the ancestral SIVgor, preventing water coordination and BHP closure (Fig. 4e). Almost all HIV-2 and SIVsmm CA sequences bear tyrosine at this position, consistent with conservation of this structural difference from pandemic HIV-1 (Fig. 4c and Supplementary Table 1). We were unable to crystallize HIV-2 CA hexamers. However, comparison of the HIV-1(O) hexamer hinge region with that of the published HIV-2 CA N-terminal domain structure (PDB ID 2 × 82)^47^ and a modelled HIV-2 hexamer illustrates the similarity between these non-pandemic capsids, and suggests an ‘open’ BHP position for HIV-2 (Fig. 4e,g). SIV from macaques (SIVmac) was unknowingly transmitted during laboratory experiments from sooty mangabeys infected with SIV sooty mangabey (SIVsmm) to rhesus macaques and is closely related to its parental SIVsmm (Fig. 4c)^48^. We solved the SIVmac hexamer structure to 2.25 Å (Fig. 4f), demonstrating conservation of the ‘open’ BHP conformation and of the hinge region, including Y50, between HIV-1(O), HIV-2 and SIVmac. This further illustrates the conformational similarity between hexamers bearing tyrosine at CA position 50 despite HIV-1(O) and SIVsmm otherwise being highly divergent (Fig. 4e–g). To probe the role of CA50 in infectivity, we made HIV-1(M) CA Q50Y, reverting the glutamine to the ancestral tyrosine. Solving the mutant hexamer structure confirmed that HIV-1(M) CA Q50Y hexamers adopted ‘open’ conformations, and the hinge region of HIV-1(M) Q50Y resembled HIV-1(O) and SIVmac (Fig. 4h). Indeed, overlay of the open WT HIV-1(M) hexamer structure (PDB ID: 5HGL) and HIV-1(M) CA Q50Y (PDB ID:7T13) demonstrated their similarity (Extended Data Fig. 5d). Importantly, and unlike other capsid point mutants, for example, CA K25A which prevents IP6 recruitment into assembling virions^49^, HIV-1(M) Q50Y capsids were not intrinsically destabilized, showing WT disassembly kinetics in the absence of IP6 in single-molecule uncoating assays (Fig. 4i and Extended Data Fig. 5e)^50^. As expected, given the similar structure, addition of IP6 led to an increase in the HIV-1(M) Q50Y capsid half-life from minutes to hours for the majority of capsids, confirming that IP6 binding was not perturbed. Nevertheless, in infection experiments, single-round infectivity of HIV-1(M) GFP CA Q50Y was reduced (Extended Data Fig. 5f) and this capsid mutant did not replicate in MDM.

### Adaptation of HIV capsid at position 120

**Fig. 5:**
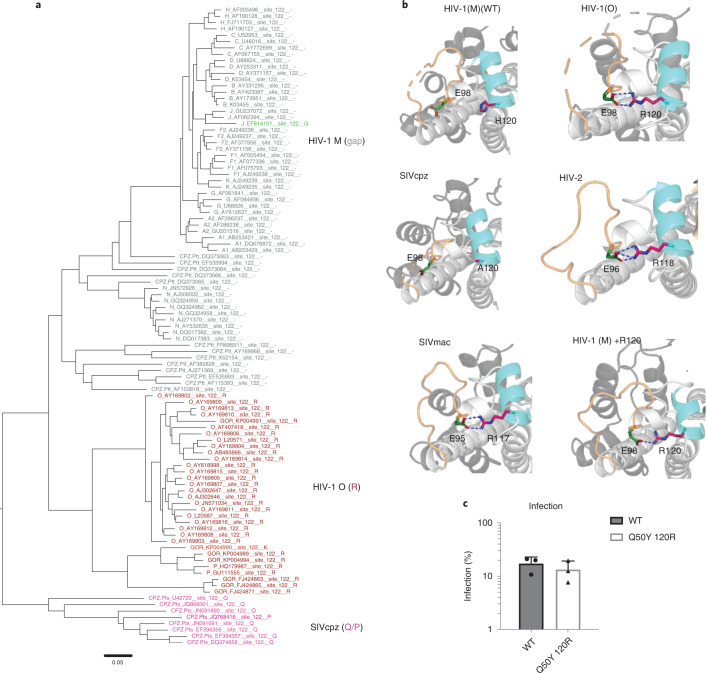
Pandemic-associated adaptation of HIV capsid at position 120. **a**, Maximum-likelihood phylogenetic tree of primate lentiviral capsid genes coloured to illustrate the residues equivalent to HIV-1(O) CA 120. Grey and (_) branch labels denote a gap in the alignment. **b**, Structures showing salt bridges in HIV-1(O) (PDB ID:7T12) (E98-R120), HIV-2 (PDB ID:2 × 82) (E96-R118), SIVmac (PDB ID:7T14) (E95-R117) and HIV-1(M) + R120 (PDB ID:7QDF) (E98-R120). The salt bridge is absent in WT HIV-1(M) CA (PDB ID:5HGN) and SIVcpz (PDB ID:7T15) because R120 is absent. Helix bearing R120 in HIV is coloured blue. Salt bridges are shown as dashed lines. CypA binding loop is coloured wheat. **c**, Single-round infection of MDM with equal genome copies of VSV-G-pseudotyped HIV-1(M) or HIV-1(M) CA Q50Y 120R GFP measured at 48 h post infection by flow cytometry. Mean ± s.e.m. *n* = 3 donors. Source data

**Extended Data Fig. 6:**
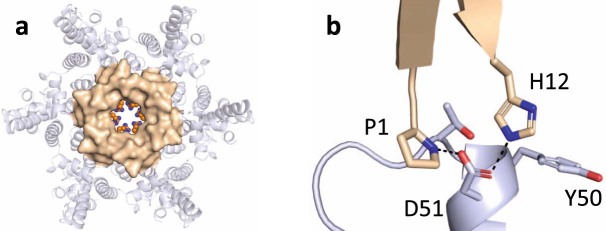
**a. Exterior view of the CA Q50Y/R120 hexamer (PDB ID:** **8D3B) showing that the beta-hairpin (wheat) adopts an open conformation.** **b**. Close-up view of the hinge region showing the H12-D51 salt-bridge necessary for the open conformation. The structure is indistinguishable from the Q50Y mutant in this region.

Given that HIV-1 CA Q50Y was defective, we returned to Chromaclade^45^ seeking additional lineage-specific changes that occurred along with capsid Y50Q in SIVcpz*Ptt*. This revealed a patch of differences around CA position 120, identifying a deletion of arginine CA 120 in HIV-1(M) and its SIVcpz*Ptt* parent (Fig. 5a). Inspection of hexamer structures from non-pandemic viruses revealed that the Arg lost from SIVcpz*Ptt*/HIV-1(M) forms a salt bridge between helix 6 and the CypA binding loop in HIV-1(O) and HIV-2 CA helix 6 (Arg 120-Glu 98 in HIV-1(O), Arg 118-Glu 96 in HIV-2) (Fig. 5b). This salt bridge is also conserved in the SIVmac hexamer (Arg 117-Glu 95), but not in the SIVcpz*Ptt* structure (Fig. 5b). These observations suggest that, in addition to Y50Q, the SIVcpz*Ptt* parent of pandemic HIV-1(M) lost a salt bridge on the CA surface through loss of an arginine. To examine its phenotypic effect, we reversed the deletion in HIV-1(M), restoring the arginine in mutant HIV-1(M) + R120. The X-ray structure of this HIV-1(M) CA + R120 hexamer mutant at 3.29 Å revealed that adding the arginine reinstated the salt bridge (Fig. 5b). An X-ray structure (PDB ID: 8D3B) of the double mutant HIV-1(M) Q50Y + R120 was poorly resolved at R120 but good resolution of the BHP and the hinge region around Y50 indicated that this mutant hexamer formed an open channel (Extended Data Fig. 6a,b). Critically combining both mutations restored infectivity of VSV-G-pseudotyped HIV-1(M) CA Q50Y + R120 in MDM (Fig. 5c).

### Reversion of adaptation renders HIV-1(M) cGAS- and TRIM5-sensitive

**Fig. 6:**
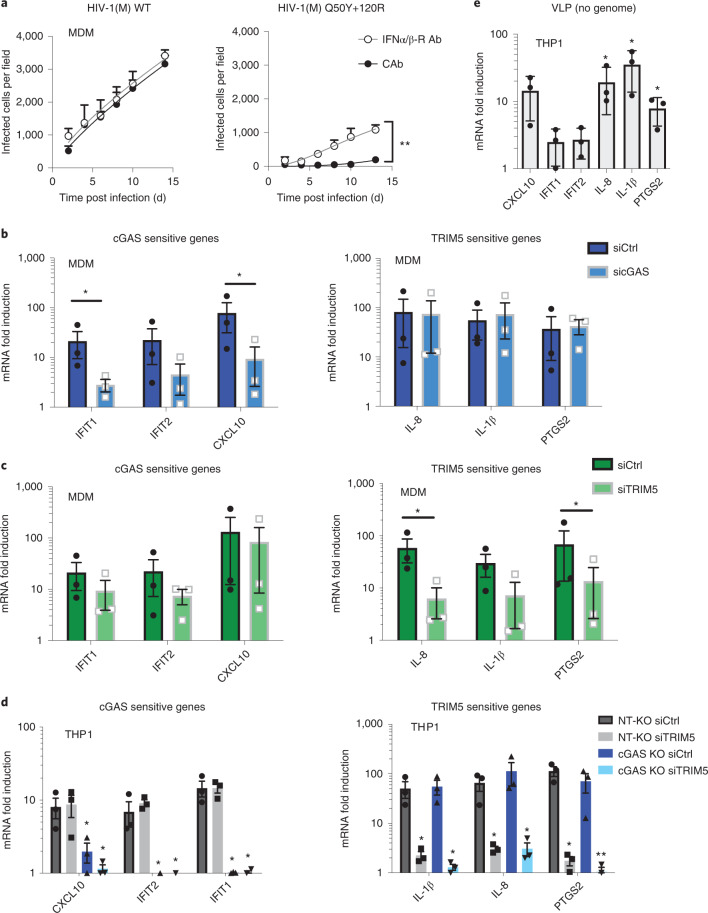
Capsid mutations make pandemic HIV-1(M) sensitive to cGAS and TRIM5. **a**, Replication of HIV-1(M) NL4.3 (BalEnv) WT or HIV-1(M) NL4.3 (BalEnv) bearing CA Q50Y + 120R in MDM in the presence of IFNα/β-R or CAb. **b**, GAPDH-normalized mRNA levels induced by HIV-1(M) CA Q50Y 120R, expressed as fold induction over uninfected samples in control siRNA-transfected (siCtrl) or cGAS siRNA-transfected (sicGAS) MDM 24 h post infection. **c**, GAPDH-normalized mRNA levels induced by HIV-1(M) CA Q50Y 120R expressed as fold induction over uninfected samples in control siRNA (siCtrl)- or TRIM5 siRNA (siTRIM5)-transfected MDM 24 h post infection. **d**, GAPDH-normalized mRNA levels induced by HIV-1(M) CA Q50Y + 120R expressed as fold induction over uninfected samples in non-targeting CRISPR-treated cells (NT-KO) transfected with control siRNA (siCtrl) (NT-KO siCtrl), NT-KO siTRIM5, cGAS KO siCtrl or cGAS KO siTRIM5 THP-1 cells measured 24 h post infection. **e**, GAPDH-normalized mRNA levels induced by HIV-1(M) Q50Y + 120R VLP (no genome) expressed as fold induction over uninfected THP-1 cells measured 24 h post infection. Mean ± s.d., *n* = 3 independent experiments or donors. Two-way ANOVA vs CAb (**a**), two-tailed unpaired *t*-test vs siCtrl (**b**,**c**), paired *t*-test vs NT-KO siCtrl THP-1 cells (**d**,**e**). *P < 0.05, **P < 0.01. Source data

**Extended Data Fig. 7:**
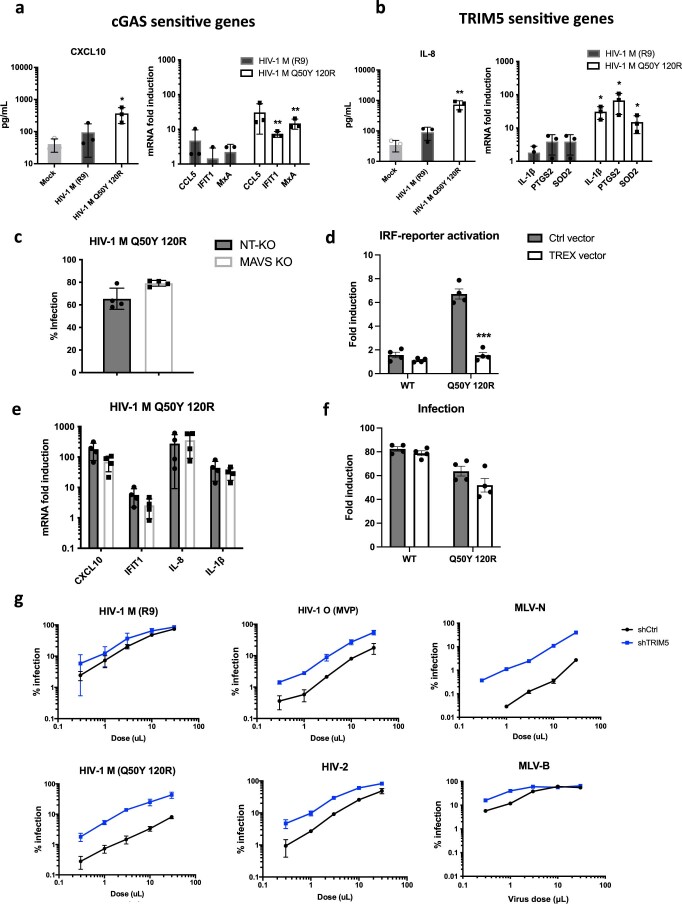
**a, b. Secreted CXCL10 (A) IL8 (B) (ELISA) 48hpi and GAPDH-normalised mRNA levels in MDM (fold induction over uninfected 24hpi).** **c**. % Infection of HIV-1(M) CA Q50Y 120 R in non-targeting CRISPR (NT-KO) or MAVS CRISPR knock out (MAVS-KO) cells. **d**. GAPDH-normalised mRNA levels, (fold induction over uninfected), in non-targeting CRISPR control (NT-KO) or MAVS KO THP-1 cells 24hpi. **e**. IRF-luciferase reporter 48 h after infection by equal genome copies of VSV-G-pseudotyped HIV-1(M) WT or Q50Y 120R mutant in ThP1 cells Ctrl or overexpressing TREX. **f**. Infection measured at 48 hours corresponding to (e) by flow. Mean + /− SD, n = 3 independent experiments or donors. **g**. VSV-G pseudotype titration curves in U87 cells expressing non-targeting control shRNA (shCtrl) or TRIM5 targeting shRNA (shTRIM5). Mean + /− SD n = 2 independent experiments. a, b two-tailed unpaired t-test vs untreated MDM. *p < 0.05, **p < 0.01. e, f paired t-test vs THP-1 Ctrl vector. ***p < 0.001. Source data

To test whether pandemic lineage-associated mutations influenced host responses, we reversed the adaptations in HIV-1(M) and infected MDM. We found that HIV-1(M) CA Q50Y + R120 could only replicate in MDM in the presence of IFNAR1-Ab, consistent with enhanced sensing and innate immune activation (Fig. 6a). Concordantly, VSV-G-pseudotyped HIV-1(M) CA Q50Y + R120 induced cGAS and TRIM5-sensitive genes in MDM more strongly than WT HIV-1(M) (Extended Data Fig. 6a,b, compare with Fig. 1e,f). cGAS-sensitive gene induction by HIV-1(M) CA Q50Y + R120 was reduced by cGAS depletion in MDM (Fig. 6b) or cGAS KO in THP-1 (Fig. 6d). TRIM5-sensitive gene induction was suppressed by TRIM5 depletion in MDM and THP-1 (Fig. 6c,d). Importantly, combined cGAS KO and TRIM5 depletion in THP-1 suppressed all gene induction by HIV-1(M) CA Q50Y+ R120 (Fig. 6d). HIV-1(M) CA Q50Y+ R120 VLPs without genome induced TRIM5-sensitive, but not cGAS-sensitive, genes in THP-1 (Fig. 6e). MAVS depletion had no effect (Extended Data Fig. 7c,d). Similarly to non-pandemic HIV, over-expression of the nuclease TREX1 in THP-1 reduced the activation of the IRF-reporter by HIV-1(M) Q50Y 120R, consistent with viral DNA exposure triggering cGAS activation (Extended Data Fig. 7e,f). Note that HIV-1(M) Q50Y 120R resembled HIV-1(O) rather that HIV-2 in that infectivity was not TREX1-sensitive. Finally, we found that HIV-1(M) CA Q50Y + R120 behaved like non-pandemic viruses HIV-1(O) and HIV-2, becoming more sensitive to human TRIM5 restriction than WT HIV-1(M), as evidenced by rescue of infection by TRIM5 depletion in U87 cells (Extended Data Fig. 7e). Infection with TRIM5-sensitive MLV-N and insensitive MLV-B acted as controls^51^. Thus, an HIV-1(M) CA mutant bearing Q50Y + R120, representing non-pandemic HIV lineages, behaved like HIV-1(O) and HIV-2, activating innate immune gene expression in a TRIM5-, cGAS- and TREX1-sensitive way. These observations outline how key amino acid adaptations in the HIV-1(M) parent SIVcpz*Ptt* led to structural changes in the capsid that reduce activation of, and restriction by, key lentiviral sensors, cGAS and TRIM5. We therefore propose that the SIVcpz*Ptt*/HIV-1(M) lineage has undergone complex adaptations that underlie its pandemic success and that this study provides mechanistic insight into how particular lineage-specific adaptations have paved the way to pandemicity.

## Discussion

HIV capsid has a pivotal role in regulating viral DNA synthesis and shielding viral DNA from cytosolic sensors. Recent work suggests that infectious fluorescently labelled intact capsids of HIV-1(M) are transported across the cytoplasm and through nuclear pores, with uncoating occurring in the nucleus before integration^15,52–55^. On the basis of the evidence in this paper, we propose that HIV-1(M) structural adaptations in capsid influence sensitivity to antiviral pathways during transport to the nucleus. For example, alteration in capsid surface dynamics mediated by loss of the Arg-Glu salt bridge may influence TRIM5 recruitment and therefore restriction and/or activation of TRIM5 signalling. Indeed, both IFN receptor blockade and TRIM5 depletion rescued infection of non-pandemic viruses in MDM (Extended Data Figs. 1 and 4e) and suppressed macrophage activation of inflammatory gene expression, suggesting important roles for both signalling and physical caging of incoming capsids by TRIM5, for antiviral activity^56^. We hypothesize that increased HIV-1(M) capsid dynamics may promote entirely encapsidated DNA synthesis by increasing core flexibility or allowing capsids to ‘breathe’, with additional co-factor-mediated regulation of the timing and position of uncoating and genome release. Indeed, TREX1 over-expression suppresses innate immune activation by both HIV-1(O) and HIV-2 and inhibits infectivity of HIV-2 because it degrades DNA from partially uncoated capsids, which are, to our surprise, infectious in the case of HIV-2 (Fig. 3). Further studies using molecular dynamic simulation in which pandemic HIV-1(M) cores are compared to non-pandemic cores may help elucidate how capsid dynamics compare and how they link to co-factor interactions and capsid stability.

Our phylogenetic analyses suggest significant complexity beyond SIVcpz*Ptt* adapting by mutating Q50Y and deleting R120 to become pandemic in humans. For example, SIVcpz*Ptt* gave rise to both pandemic HIV-1(M), which retained Q50, and non-pandemic HIV-1(N), which only infected a handful of people but experienced strong selection at Q50 making Q50S, Q50A or Q50G, each requiring multiple nucleotide changes (Fig. 5). On the other hand, SIVsmm/SIVgor/HIV-2 retained Y50 in chimpanzee-to-gorilla and gorilla-to-human HIV-1(O) zoonoses (Fig. 5). Furthermore, the SIVcpz*Ptt* progenitor also gave rise to viruses retaining the ancestral tyrosine at CA50 (SIVgor, HIV-1(O)), suggesting an unsampled SIVcpz*Ptt* lineage that retained this amino acid. Further evidence against a simple model is derived from our failure to increase HIV-2/HIV-1(O) replication in human MDM by mutating CA Y50Q, although we did not try combinations of mutations, for example, additionally deleting R120. While we cannot identify the selective pressures that selected capsid adaptations, we propose that complexity arises from the diversity of capsid function and the species-specific co-factor interactions that govern regulation of DNA synthesis, uncoating and nuclear entry. Future studies applying chromaclade-guided mutagenesis may reveal how capsids work, linking dynamics to co-factor use, myeloid cell replication and pandemicity.

HIV-2 and HIV-1(O) replicate efficiently in activated primary human T cells in vitro^35,57,58^. Here we focused on primary human macrophages because primary T cells are not permissive to HIV unless activated, typically by cross-linking the T-cell receptor (TCR) to mimic antigen stimulation. Thus, T-cell receptor-driven signalling and cytokine secretion dominate in vitro T-cell infection experiments, obviating virus-induced changes including cGAS/TRIM5 activation. Furthermore, macrophages effectively secrete type-I IFN that can inhibit lentiviral transmission^30^ and is present during transmission-induced cytokine storms^59^. An important role for IFN in HIV transmission is also evidenced by the unique resistance of transmitted founder HIV-1 to type-I interferons^60^.

Together with our data, these previous observations emphasize the importance of innate immune evasion as a key determinant of transmission and therefore pandemic potential. We note that enhanced evasion of innate sensing occurred in viruses infecting central chimpanzees (*Pan troglodytes troglodytes*) before transmission in humans, suggesting that SIVcpz*Ptt* are more dangerous to humans than SIVcpz*Pts* strains infecting eastern chimpanzees (*Pan troglodytes schweinfurthii*), which have never been detected in the human population. This in turn suggests that we may be able to predict zoonosis-competent viruses by examining their capacity to escape human innate immunity. We note that the first detected SARS-CoV-2 isolate antagonized human innate immunity effectively despite bat origins, whereas more recently evolved variants have increased innate immune evasion capabilities, again linking innate immune evasion to increased human-to-human transmission^61^.

We propose that a detailed understanding of the innate immune mechanisms that protect us from zoonosis, and a better understanding of how pandemic viruses evolve to avoid these defences, will be crucial for future pandemic preparedness. In this respect, HIV is a very well-understood virus that offers excellent tools for further relevant discoveries in this field.

## Methods

### Cells and reagents

HEK293T and U87 cells were maintained in DMEM medium (Gibco) supplemented with 10% fetal bovine serum (FBS, Labtech) and 100 U ml^−1^ penicillin plus 100 μg ml^−1^ streptomycin (Pen/Strep; Gibco). THP-1-IFIT1 cells that had been modified to express *Gaussia* luciferase under the control of the *IFIT1* promoter were described previously^62^. THP-1 dual control and cGAS^−/−^ cells were obtained from Invivogen. THP-1 IFIT1 cells were maintained in RPMI medium (Gibco) supplemented with 10% FBS and Pen/Strep. THP-1 dual cells were maintained in RPMI (Gibco) supplemented with 10% FBS, Pen/Strep, 25 mM HEPES (Sigma), 10 µg ml^−1^ of blasticidin (Invivogen) and 100 μg ml^−1^ of Zeocin (Invivogen). GHOST cells stably expressing CD4, CCR5, CXCR4 and the green fluorescent protein (GFP) reporter gene under the control of the HIV-2 long terminal repeat, were maintained in DMEM supplemented with 10% FBS, and antibiotics, G418 (500 μg ml^−1^) (Thermo Fisher), hygromycin (100 μg ml^−1^)(Invitrogen) and puromycin (1 μg ml^−1^) (Millipore). Lipopolysaccharide, IFNβ, IL-1β and poly I:C were obtained from Peprotech. Herring testes (HT) DNA was obtained from Sigma. For stimulation of cells by transfection, transfection mixes were prepared using lipofectamine 2000 (Invitrogen) in Optimem (Thermo Fisher). HT DNA and poly I:C concentration used are stated on each figure.

### Isolation of primary MDM

Primary MDM were prepared from fresh blood from healthy volunteers. The study was approved by the joint University College London/University College London Hospitals NHS Trust Human Research Ethics Committee and written informed consent was obtained from all participants.

Peripheral blood mononuclear cells (PBMCs) were isolated by density-gradient centrifugation using Lymphoprep (Stemcell Technologies), washed three times (PBS) and plated to select for adherent cells. Non-adherent cells were washed away after 1.5 h and the remaining cells incubated in RPMI (Gibco) supplemented with 10% heat-inactivated pooled human serum (Sigma) and 100 ng ml^−1^ macrophage colony stimulating factor (R&D systems). For replication experiments with full-length viruses, the medium was then refreshed after 3 d (RPMI 1640 with 10% human serum), removing any remaining non-adherent cells. After 6 d, media were replenished with RPMI containing 5% type AB human serum (Sigma-Aldrich). For single-round experiments with VSV-G-pseudotyped viruses, cells were washed (PBS) on day 3 of differentiation and the medium changed to RPMI supplemented with 10% heat-inactivated FBS. MDM were then infected 3–4 d later. Replicate experiments were performed with cells derived from different donors.

### Editing of cells by CRISPR/Cas9

Lentiviral particles to generate CRISPR/Cas9-edited cell lines were produced by transfecting 10 cm dishes of HEK293T cells with 1.5 μg of pLentiCRISPRv2 encoding gene-specific guide RNAs (Addgene plasmid 52961), 1 μg of p8.91 packaging plasmid^40^ and 1 μg of VSV-G glycoprotein-expressing plasmid pMDG (Genscript) using Fugene-6 transfection reagent (Promega). Virus supernatants were collected at 48 and 72 h post transfection, pooled and used to transduce THP-1 IFIT1 cells by spinoculation (1,000 × *g*, 1 h, room temperature). Transduced cells were selected using puromycin (1 μg ml^−1^, Merck Millipore) and single clones isolated by limiting dilution in 96-well plates. Clones were screened for successful gene knock out by luciferase assay after targeted protein stimulation and immunoblotting.

gRNA sequences:

MAVS: CAGGGAACCGGGACACCCTC

Non-targeting control: ACGGAGGCTAAGCGTCGCAA

### Virus plasmids

The NL4.3 (Ba-L Env), YU2 (ref. ^31^) and O-group molecular clones, RBF206 and BCF120, and HIV-2 molecular clones pJK7312S^3^ and pST^63^ have all been described. HIV-2 ROD10 was obtained from the National Institute of Biological Standards and Controls^64^. The CA chimera molecular clone was generated by overlap PCR, replacing CA residues 1–204 of NL4.3 with the equivalent residues from MVP5180 or HIV-2 ROD10. VSV-G-pseudotyped GFP-encoding vectors include HIV-1 M LAI ΔEnv.GFP (LAI strain^39^) with the Nef coding region replaced by GFP, HIV-1(M) R9 packaging vector (p8.91) and minimum genome-expressing GFP (CSGW)^41^. HIV-2 ROD GFP has been described^65^. HIV-1(O) packaging plasmid to make HIV-1(O) GFP was generated by replacing Gag-Pro residues between *Not*1–*Bcl*1 in p8.91 with the equivalent residues from MVP5180 (ref. ^66^). Q50Y and 120R mutations were generated by site directed mutagenesis of p8.91 using *Pfu*Turbo (Agilent Technologies). For TREX1 over-expression, we used MLV-based gammaretroviral expression vector EXN^67^, where TREX1 coding sequence was cloned from a plasmid kindly provided by Nan Yan between *BamHI* and *XhoI* sites. For TRIM5 depletion with short hairpin (shRNA), we expressed shRNA using SIREN-RetroQ (Clontech) gammaretroviral vector containing shRNA sequence targeting human TRIM5 (ref. ^26^) or scramble Ctrl^29^ as described.

### Production of virus in HEK293T cells

Replication competent HIV were produced by transfection of HEK293T cells in T150 flasks using Fugene-6 transfection reagent (Promega). Briefly, just-subconfluent T150 flasks were transfected with 8.75 μg of vector and 30 µl Fugene-6 in 500 µl Optimem (Thermo Fisher). Virus supernatants were collected at 48, 72 and 96 h post transfection. Virus suspensions were filtered, subjected to ultracentrifugation through a 20% sucrose buffer and resuspended in RPMI 1640 with 5% human serum for subsequent replication experiments in MDM. For VSV-G-pseudotyped GFP-expressing virus, each T150 flask was transfected with 2.5 μg of VSV-G glycoprotein-expressing plasmid pMDG (Genscript) and 6.25 μg of pLAIΔEnv GFP or 2.5 μg packaging plasmid (p8.91, MVP or HIV-2-pack) and 3.75 μg of GFP-encoding genome plasmid (CSGW or HIV-2 GFP) using 30 µl Fugene-6 in 500 µl Optimem. In the case of VLP without genome, the cells in T150 flasks were transfected only with 2.5 μg of VSV-G glycoprotein-expressing plasmid pMDG and 5 μg packaging plasmid (p8.91, MVP or HIV-2). Virus supernatants were collected at 48 and 72 h post transfection, pooled, DNase treated (2 h at 37 °C, DNaseI, Sigma) and subjected to ultracentrifugation over a 20% sucrose cushion. Viral particles were finally resuspended in RPMI supplemented with 10% FBS. Lentiviral particles to generate TREX-expressing vector or TRIM5 shRNA vector were produced by transfecting 10 cm dishes of HEK293T cells with 1.5 μg TREX EXN vector or TRIM5-targeting SIREN-RetroQ, 1 μg of packaging plasmid CMVintron, and 1 μg of VSV-G glycoprotein-expressing plasmid pMDG using Fugene-6 transfection reagent. Virus supernatants were collected at 48 and 72 h post transfection, pooled and stored at −80 °C.

### Virus quantification and RT products

Full-length HIV clones were quantified by RT enzyme-linked immunosorbent assay (ELISA) (Roche). Reverse transcriptase activity of virus preparations was quantified by qPCR using a SYBR Green-based product-enhanced RT (SG-PERT) assay as previously described^68^. For viral genome copy measurements, RNA was extracted from 2 μl sucrose-purified virus using the RNeasy mini kit (QIAGEN). The RNA was then treated with TURBO DNase (Thermo Fisher) and subjected to reverse transcription using Superscript III reverse transcriptase and random hexamers (Invitrogen). Genome copies were then measured by *Taq*man qPCR using primers against GFP^69^ (see below).

For RT product measurements, DNA was extracted from 5 × 10^5^ infected cells using the DNeasy blood and tissue kit (QIAGEN). DNA concentration was quantified using a Nanodrop for normalization. RT products were quantified by *Taq*man qPCR using *Taq*Man gene expression master mix (Thermo Fisher) and primers and probe specific to GFP. A dilution series of plasmid encoding GFP was measured in parallel to generate a standard curve to calculate the number of GFP copies.

Primers:

*GFP* fwd: 5′- CAACAGCCACAACGTCTATATCAT -3′

*GFP* rev: 5′- ATGTTGTGGCGGATCTTGAAG -3′

*GFP* probe: 5′- FAM-CCGACAAGCAGAAGAACGGCATCAA-TAMRA -3′

### Infection assays

To measure viral replication, MDM were infected with 100 pg RT of full-length viruses, measured by RT ELISA (Roche) per well (multiplicity of infection (MOI) = 0.2) in 48-well plates and subsequently fixed and stained using mixed CA antibodies EVA365 and EVA366 (National Institute of Biological Standards AIDS Reagents) at 1/50, with goat anti-mouse immunoglobulin (Ig) antibody conjugated to β-galactosidase (926-32210, Southern Biotechnology Associates) at 1/15,000, and counted^31^. Anti-IFN-α/β receptor (PBL Interferon Source) or control IgG2A antibody (R&D systems) were added at 1 μg ml^−1^ for 2 h before infection and supplemented every 4 d. Single-round infection by VSV-G-pseudotyped viruses was performed in 48-well plates using equal viral doses (1 × 10^9^ genome copies). Viral infection was measured 48 h later by enumeration of GFP-positive cells by flow cytometry. For RNA extraction and subsequent qPCR analysis, cells were infected in 24-well plates.

GHOST cells were infected with full-length viruses as previously described^31^, measured by RT ELISA per well in 48-well plates. Cells were fixed at the indicated times post infection and GFP+ cells measured by flow cytometry.

Monocytic THP-1 cells were infected at a density of 2 × 10^5^ cells per ml in 48-well plates in the presence of polybrene (8 μg ml^−1^, Sigma). Infection levels were assessed at 48 h post infection through enumeration of GFP-positive cells by flow cytometry. Input dose of virus was normalized either by RT activity (measured by SG-PERT) or genome copies (measured by qPCR) as indicated. THP-1 cells were treated with similar doses of VLP normalized by SG-PERT as indicated. After 24 h, cells were infected with equal amounts of genome copies (2 × 10^8^) and infection levels were measured 48 h post infection through enumeration of GFP-positive cells by flow cytometry.

THP-1 cells stably expressing TREX were generated by transduction with the MLV-based gammaretroviral expression vector EXN and maintained under selection with G418 (500 μg ml^−1^).

IFNβ (100 ng ml^−1^) or IL-1β (10 ng ml^−1^) were added at different time points to THP-1 cells, which were then infected at an MOI of 0.3. Infection levels were measured after 48 h by flow cytometry.

### Luciferase and secreted alkaline phosphatase reporter assays

*Gaussia*/*Lucia* luciferase activity was measured by transferring 10 μl supernatant to a white 96-well assay plate, injecting 50 μl per well of coelenterazine substrate (Nanolight Technologies, 2 μg ml^−1^) and analysing luminescence on a FLUOstar OPTIMA luminometer (Promega). Data were normalized to a mock-treated control to generate a fold induction. Secreted alkaline phosphatase was measured using QUANTI-Blue (Invivogen), using 20 µl of cell supernatant.

### Quantitative RT-PCR

RNA was extracted from MDM or THP-1 cells using RNAeasy (QIAGEN). RNA (500 ng) was used to synthesize complementary DNA using Superscript III reverse transcriptase (Invitrogen). cDNA was diluted 1:5 in water and 2 μl was used as a template for real-time PCR using SYBR Green PCR master mix (Applied Biosystems) and QuantiStudio 5 real-time PCR machine (Applied Biosystems). Expression of each gene was normalized to an internal control (*GAPDH*) and values were then normalized to mock-treated control cells to yield a fold induction. Primers:

*GAPDH:* Fwd 5′-GGGAAACTGTGGCGTGAT-3′, Rev 5′-GGAGGAGTGGGTGTCGCTGTT-3′

*CXCL10:* Fwd 5′-TGGCATTCAAGGAGTACCTC-3′, Rev 5′-TTGTAGCAATGATCTCAACACG-3′

*IFIT2:* Fwd 5′-CAGCTGAGAATTGCACTGCAA-3′, Rev 5′-CGTAGGCTGCTCTCCAAGGA-3′

*MxA:* Fwd 5′-ATCCTGGGATTTTGGGGCTT-3′, Rev 5′-CCGCTTGTCGCTGGTGTCG-3′

*CCL5:* Fwd: 5′-CCCAGCAGTCGTCTTTGTCA-3′, Rev 5′- TCCCGAACCCATTTCTTCTCT-3′

*IFIT1:* Fwd: 5′- CCTCCTTGGGTTCGTCTACA-3′, Rev 5′-GGCTGATATCTGGGTGCCTA-3′

*IL-8:* Fwd: 5′-ATGACTTCCAAGCTGGCCGTGGCT-3′, Rev 5′-TCTCAGCCCTCTTCAAAAACTTCTC-3′*PTGS2:* Fwd: 5′-CTGGCGCTCAGCCATACAG-3′, Rev 5′-CGCACTTATACTGGTCAAATCCC-3′

*IL-1β:* Fwd: 5′-ATGATGGCTTATTACAGTGGCAA-3′, Rev 5′-GTCGGAGATTCGTAGCTGGA-3′

*SOD2:* Fwd: 5′-GGAAGCCATCAAACGTGACTT-3′, Rev 5′-CCCGTTCCTTATTGAAACCAAGC-3′

*cGAS:* Fwd 5′*-*GGGAGCCCTGCTGTAACACTTCTTAT-3′ Rev, 5′-TTTGCATGCTTGGGTACAAGGT-3′

*TREX:* Fwd 5′*-*CGCATGGGCGTCAATGTTTT-3′ Rev, 5′-GCAGTGATGCTATCCACACAGAA-3′

TRIM5 expression levels were measured using *Taq*Man gene expression assay detecting TRIM5 (FAM dye-labelled, *Taq*Man probe Hs01552559_m1), or the housekeeping gene OAZ1 (FAM dye-labelled, primer-limited, *Taq*Man probe Hs00427923_m1).

### ELISA

Cell supernatants were collected for ELISA at 48 h post infection/stimulation and stored at −20 °C. CXCL10 and IL-8 protein were measured using Duoset ELISA reagents (R&D Biosystems).

### cGAS and TRIM5 depletion by RNAi

MDM (1 × 10^5^) differentiated in macrophage colony stimulating factor for 4 d were transfected with 25 pmol of siRNA SMART pool against cGAS (L-015607-02-0005), TRIM5 (L-007100-00-0005) or non-targeting control (D-001810-10-05) (Dharmacon) using lipofectamine RNAiMAX transfection reagent (Invitrogen). Medium was replaced after 18 h with RPMI 1640 supplemented with 10% FCS and cells cultured for an additional 3 d before infection. THP-1 dual cells (5 × 10^5^ ml^−1^) were transfected with 35 pmol of siRNA SMART pool against cGAS, TRIM5 or non-targeting control (Dharmacon) using lipofectamine RNAiMAX (Invitrogen). Medium was replaced after 18 h with RPMI 1640 supplemented with 10% FCS and cells were plated in 48-well plates and infected as indicated. To deplete TRIM5 in U87 cells, pSIREN-RetroQ expressing shRNA TRIM5 was transduced at an MOI ∼1 and shRNA-expressing cells selected with 10 μg ml^−1^ puromycin. TRIM5 and cGAS expression were quantified by qPCR normalized to OAZ1 and GAPDH, respectively, by the delta delta threshold cycle (ΔΔCt) method.

### Immunoblotting

Cells were lysed in 50 mM Tris buffer (pH 8), 150 mM NaCl, 1 mM EDTA, 10% (v/v) glycerol, 1% (v/v) Triton X-100, 0.05% (v/v) NP40 supplemented with protease inhibitors (Roche), clarified by centrifugation (14,000 × *g* for 10 min), and the supernatants boiled (5 min in 6X Laemmli buffer (50 mM Tris-HCl (pH 6.8), 2% (w/v) SDS, 10% (v/v) glycerol, 0.1% (w/v) bromophenol blue, 100 mM β-mercaptoethanol)) before separation on 12% polyacrylamide gel. Proteins were transferred to Hybond ECL membrane (Amersham Biosciences) using a semi-dry transfer (Biorad). Primary antibodies goat anti-MAVS Ab (Cell Signaling, 3993, 1:1,000 dilution) and goat anti-tubulin Ab (Abcam, ab6046, 1:20,000 dilution) were detected with IRDye 800CW goat anti-rabbit IgG (H + L) (LI-COR, 926-32211, 1:20,000) and membranes imaged with an Odyssey CLX infrared imager (LI-COR Biosciences) using Image Studio V5.2.

### Single-molecule analysis

Single-particle traces of AF488-CypA-labelled capsids inside permeabilized virions immobilized on a coverslip were recorded by total internal reflection (TIRF) microscopy and analysed by step fitting to determine distributions of capsid lifetimes for WT and mutant HIV^50^.

### Phylogenetics

A dataset of representative HIV and SIV sequences from the CA region of *gag* were downloaded from the Los Alamos HIV-1 sequence database and aligned manually. The phylogeny was estimated from the nucleotide sequences using RAxML v8 (ref. ^70^) with substitution model GTR+ Gamma and rooted consistent with phylogenies that include non-primate lentivirus outgroup taxa^71^. ChromaClade v1.1 was used to annotate taxon labels with residues found at capsid protein sites^45^. Note that chromaclade does not use statistical tests to assess viral evolution. Rather, it provides a simple way to visualize lineage-specific amino acid variation in a qualitative and intuitive way. In this study, our focus on positions CA50 and 120 was also influenced by the hexamer structures, which revealed that these positions have a role in the structural differences observed between viral capsid hexamers.

### Protein production and purification

#### HIV-1(M) CA R120

Protein was expressed and purified as previously described for HIV-1(M) CA WT^72^. In brief, HIV-1(M) CA R120 was expressed in *E. coli* C41 OverExpress C41(DE3) (Lucigen) in 1 l 2YT media supplemented with 100 μg ml^−1^ ampicillin at 37 °C and 250 r.p.m. until optical density at 600 nm reached 0.5, followed by the addition of 0.4 mM isopropyl β-D-1-thiogalactopyranoside (IPTG) to induce expression overnight at 14 °C. Cells were lysed in 50 mM Tris-HCl, 40 mM NaCl and 20 mM β-mercaptoethanol (pH 4.5) using a cell disruptor, followed by removal of the insoluble fraction (30,000 × *g* for 20 min). Capsid protein was precipitated with 20% (w/v) ammonium sulfate and pelleted (30,000 × *g* for 20 min). Pellets were resuspended in refolding buffer (100 mM citric acid, 20 mM TRIS (pH 4.5)), followed by extensive dialysis against the same buffer and then 25 mM Tris-HCl (pH 8). The capsid was further purified with anion exchange chromatography (AEC) using a 5 ml Hi-TRAP Q column (Cytiva). AEC purification was performed using buffer A (25 mM Tris-HCl (pH 8)) and buffer B (25 mM Tris-HCl, 1 M NaCl (pH 8)). Lastly, size exclusion chromatography (SEC) was performed using a Superdex 16/600 75 pg column with 25 mM Tris-HCl and 40 mM NaCl (pH 8). The protein was further concentrated to 3.0 mg ml^−1^ in the size exclusion chromatography buffer for crystallization.

#### HIV-1(O), HIV-1(M) CA Q50Y, SIVmac, SIVcpz*Ptt*

Hexameric CA proteins, stabilized by engineered inter-subunit disulfide bonds, were produced by assembly of recombinant CA containing four amino acid substitutions^73^: HIV-1(O-group) (A14C, E45C, W185A, M186A); HIV-1(M-group) (A14C, E45C, Q50Y, W184A, M185A); SIVmac (P13C, E44C, W182A, M183A); and SIVcpz (P14C, E45C, W184A, M185A). Expression was performed in *E. coli* (C41) by mid-log induction with 1 mM IPTG^14^ overnight at 14 °C. Collected cells were lysed in 50 mM Tris (pH 8.0), 150 mM NaCl and 20 mM β-mercaptoethanol by sonication. Clarified lysates were treated with 20% (w/v) ammonium sulfate and the precipitate resuspended in 100 mM citric acid (pH 4.5) and 20 mM β-mercaptoethanol, and dialysed against the same to remove the ammonium sulfate. Redissolved protein was subjected to assembly by three dialysis steps: (1) 1 M NaCl, 50 mM Tris (pH 8.0), 20 mM β-mercaptoethanol; (2) 1 M NaCl, 50 mM Tris (pH 8.0); and (3) 20 mM Tris (pH 8.0), 40 mM NaCl. Purified hexamers were isolated by size exclusion chromatography using a 16/600 Superdex 200 Prep Grade column on an ÄKTA Pure with 20 mM Tris and 40 mM NaCl.

### Crystallization, structure solution and analysis

#### HIV-1(M) CA R120

Crystals were grown using the hanging-drop vapour-diffusion technique at 20 °C by mixing 1 μl protein with 1 or 2 ul precipitant containing 9.5–11% (w/v) PEG3350, 250–350 mM NaI and 100 mM sodium cacodylate (pH 6.5) as previously described^74^. Collected crystals were immersed in precipitant mixture with 20% (v/v) glycerol and cryo-cooled in liquid nitrogen. Diffraction data were collected from a single crystal at the PETRA III P13 beamline (EMBL Hamburg/DESY P13, Germany). The dataset was indexed, processed and scaled using XDS vJan31,2020 (ref. ^75^). The HIV-1(M) CA R120 crystal belonged to the P6 space group with a solvent content of 48.5% corresponding to one molecule per asymmetric unit. The structure was determined by molecular replacement using Phenix Phaser v2.8.3 (ref. ^76^) and a previously determined HIV-1(M) CA structure (PDB ID:4XFX) as search model. Model building was performed using COOT v0.8.9.2 (ref. ^77^). Refinement was performed using REFMAC v5.8. Overview of refinement procedures was within REFMAC5: utilizing data from different sources^78^ using a TLS/maximum-likelihood protocol. The model converged to a final Rwork/Rfree of 0.242/0.277 at a resolution of 2.30 Å. The HIV-1(M) CA R120 model covers the HIV-1(M) CA amino acid sequence 1–222 and contains in addition 2 iodine, 4 chlorine ions and 14 water molecules. Figures were rendered using PyMOL (version 2.5.0.a0, Schrodinger).

#### HIV-1(O), HIV-1(M) CA Q50Y, HIV-1(M) CA Q50Y/R120, SIVmac, SIVcpz

HIV-1(O-group) hexamer crystals were grown using hanging-drop vapour-diffusion with 2 μl protein (40 mg ml^−1^) +2 μl crystallant (10% (w/v) PEG 6000, 100 mM HEPES (pH 7.0), 100 mM glycine) suspended over 500 μl undiluted crystallant. Crystals were cryoprotected with the gradual addition of glucose (solid) to 40% (w/v). HIV-1(M-group, Q50Y) hexamer crystals were grown using hanging-drop vapour-diffusion with 2 μl protein (13 mg ml^−1^) +2 μl crystallant (19% (v/v) PEG 550MME, 100 mM Tris (pH 8.0), 150 mM KSCN, 10 mM ATP, 3% (v/v) 3,5-hexanediol) suspended over 500 μl undiluted crystallant and cryoprotected in 20% (v/v) 2-methyl-2,4-pentanediol (MPD). HIV-1(M-group, Q50Y/R120) hexamer crystals were grouped using sitting-drop vapour-diffusion with 1 μl protein (12 mg ml^−1^) +1 μl crystallant (20% PEG 550MME, 0.1 M Tris (pH 8.0), 0.15 M KSCN, 10 mM ATP, 3% ethanol) suspended over 80 μl crystallant and cryoprotected in 20% (v/v) MPD. SIVmac hexamer crystals were grown using sitting-drop vapour-diffusion with 200 nl protein (12 mg ml^−1^) +200 nl crystallant (10% (w/v) PEG 6000, 5% (w/v) MPD, 100 mM HEPES (pH 7.5)) suspended over 80 μl crystallant and cryoprotected in 20% (v/v) MPD. SIVcpz hexamer crystals were grown using sitting-drop vapour-diffusion using 200 nl protein (12 mg ml^−1^) +200 nl crystallant (4.5% PEG 550MME, 0.15 M KSCN, 0.1 M Tris (pH 9.0), 4% 2,5-hexanediol) suspended over 80 μl crystallant and cryoprotected in 20% (v/v) MPD. Diffraction data were collected at 100 K on Diamond Light Source beamlines I02 (HIV-1(O), HIV-1(M), Q50Y) and I04-1 (SIVmac, SIVcpz) or in-house (M-group Q50Y/R120) on a Rigaku FR-E Superbright rotating anode source equipped with an MAR345 image plate detector. Data were reduced using IMOSFLM v7.4 (ref. ^79^) or XDS v0.6.5.2 (ref. ^75^), and scaled and merged using AIMLESS v0.7.4 (ref. ^80^). Structures were solved by molecular replacement using PHASER v2.8.3 (ref. ^81^) and search model based on the original cross-linked HIV-1(M) hexamer, PDB:3H47 (ref. ^73^). Structures were refined using REFMAC5 v5.8 (ref. ^82^) or phenix.refine v1.17.1.3660 (ref. ^83^). Between rounds of refinement, models were manually checked and corrected against the corresponding electron-density maps in COOT^84^. The quality of the model was regularly checked for steric clashes, incorrect stereochemistry and rotamer outliers using MOLPROBITY v4.02b-467Xtriage^85^.

### Position-specific scoring matrices (PSSMs)

Sequence alignments for the capsids of HIV-1(M), HIV-1(O), HIV-2, SIVcpz*Ptt*_and SIVcpz*Pts*_ were either obtained as pre-made alignments from the LANL HIV Database, or directly from NCBI Virus followed by multiple sequence alignment using Clustal Omega. All alignments were manually adjusted, and sequences with larger insertions, deletions and/or poor sequence coverage were excluded. PSSMs (Supplementary Table 1) were generated using an R-script provided by Julian Villabona Arenas.

### Statistical analysis

We have included the number of replicates (equal to the number of different donors), statistical tests and significance criteria in figure legends and in the main text. Statistical analysis was performed in GraphPad Prism. The following *P* values were considered significant: ***P ≤ 0.001, **P ≤ 0.01, *P ≤ 0.05. Data collection and refinement statistics of the protein structures solved in this paper can be found in Supplementary Table 2.

### Reporting summary

Further information on research design is available in the Nature Research Reporting Summary linked to this article.

## Supplementary information

Reporting Summary

Supplementary Table 1 PSSM tables.

Supplementary Table 2 Structure data collection and refinement statistics.

## Data Availability

The PDB numbers for the new structures solved in this paper are: HIV-1(M) R120: 7QDF; HIV-1(O) Hexamer: 7T12; HIV-1(M) Q50Y Hexamer: 7T13; SIVmac Hexamer: 7T14; SIVcpz: 7T15; HIV-1(M) Q50Y 120R Hexamer: 8D3B. The rest of the data that support the findings of this study can be found in the supplementary information as source data or are available from the corresponding authors upon request. Source data are provided with this paper.

## Source data

Source Data Fig. 1 Raw data supporting graphs.
Source Data Fig. 2 Raw data supporting graphs.
Source Data Fig. 3 Raw data supporting graphs.
Source Data Fig. 4 Raw data supporting graphs.
Source Data Fig. 5 Raw data supporting graphs.
Source Data Fig. 6 Raw data supporting graphs.
Source Data Extended Data Fig. 1 Raw data supporting graphs.
Source Data Extended Data Fig. 2 Raw data supporting graphs.
Source Data Extended Data Fig. 2 Raw MAVS western blot.
Source Data Extended Data Fig. 3 Raw data supporting graphs.
Source Data Extended Data Fig. 4 Raw data supporting graphs.
Source Data Extended Data Fig. 5 Raw data supporting graphs.
Source Data Extended Data Fig. 7 Raw data supporting graphs.
