# Supplementary material for: Evasion of cGAS and TRIM5 defines pandemic HIV
Source: Nat Microbiol. 2022 Oct 26;7(11):1762–76. doi: 10.1038/s41564-022-01247-0 (PMC9613477; doi:10.1038/s41564-022-01247-0)
Supplement: Supplementary file 1 — Reporting Summary [file 41564_2022_1247_MOESM1_ESM.pdf]

nature portfolio

Corresponding author(s): Greg Tower
David Jacques

Last updated by author(s): Aug 19, 2022

Reporting Summary

Nature Portfolio wishes to improve the reproducibility of the work that we publish. This form provides structure for consistency and transparency in reporting. For further information on Nature Portfolio policies, see our Editorial Policies and the Editorial Policy Checklist.

Statistics

For all statistical analyses, confirm that the following items are present in the figure legend, table legend, main text, or Methods section.

n/a	Confirmed	
☐	☒	The exact sample size (n) for each experimental group/condition, given as a discrete number and unit of measurement
☐	☒	A statement on whether measurements were taken from distinct samples or whether the same sample was measured repeatedly
☐	☒	The statistical test(s) used AND whether they are one- or two-sided<br>*Only common tests should be described solely by name; describe more complex techniques in the Methods section.*
☒	☐	A description of all covariates tested
☐	☒	A description of any assumptions or corrections, such as tests of normality and adjustment for multiple comparisons
☐	☒	A full description of the statistical parameters including central tendency (e.g. means) or other basic estimates (e.g. regression coefficient) AND variation (e.g. standard deviation) or associated estimates of uncertainty (e.g. confidence intervals)
☐	☒	For null hypothesis testing, the test statistic (e.g. F, t, r) with confidence intervals, effect sizes, degrees of freedom and P value noted<br>*Give P values as exact values whenever suitable.*
☒	☐	For Bayesian analysis, information on the choice of priors and Markov chain Monte Carlo settings
☒	☐	For hierarchical and complex designs, identification of the appropriate level for tests and full reporting of outcomes
☐	☒	Estimates of effect sizes (e.g. Cohen's d, Pearson's r), indicating how they were calculated

Our web collection on statistics for biologists contains articles on many of the points above.

Software and code

Policy information about availability of computer code

Data collection	Image Studio v5.2 (blots), QuantStudio v5 (QPCR)
Data analysis	As outlined in methods section we used XDS (0.6.5.2)<br>Cootv1<br>IMOSFLM (7.4.0)<br>AIMLESS (0.7.4)<br>Phenix Phaser (2.8.3)<br>Phenix.refine (1.17.1.3660)<br>REFMAC (5.8)<br>AIMLESS (0.7.4)<br>MOLPROBITY (4.02b-467Xtriage)<br>PyMOL Molecular Graphics System, Version 2.5.0.a0 Schrodinger, LLC.<br>Chromaclade v1.1<br>RAxML v8<br>XDS Jan31,2020

For manuscripts utilizing custom algorithms or software that are central to the research but not yet described in published literature, software must be made available to editors and reviewers. We strongly encourage code deposition in a community repository (e.g. GitHub). See the Nature Portfolio guidelines for submitting code & software for further information.

Data

Policy information about availability of data

All manuscripts must include a data availability statement. This statement should provide the following information, where applicable:

- Accession codes, unique identifiers, or web links for publicly available datasets
- A description of any restrictions on data availability
- For clinical datasets or third party data, please ensure that the statement adheres to our policy

The PDB numbers for the new structures solved in this paper are: HIV-1(M) R120: 7QDF; HIV-1(O) Hexamer: 7T12; HIV-1(M) Q50Y Hexamer: 7T13; SIVmac Hexamer: 7T14; SIVcpz: 7T15; HIV-1 (M) Q50Y 120R Hexamer: 8D3B. The rest of the data that support the findings of this study can be found in the supplementary information as source data or are available from the corresponding author upon request.

Field-specific reporting

Please select the one below that is the best fit for your research. If you are not sure, read the appropriate sections before making your selection.

- [x] Life sciences
- [ ] Behavioural & social sciences
- [ ] Ecological, evolutionary & environmental sciences

For a reference copy of the document with all sections, see nature.com/documents/nr-reporting-summary-flat.pdf

Life sciences study design

All studies must disclose on these points even when the disclosure is negative.

Sample size	Sample sizes for biological replicates (provided in the figure legends) were determined following pilot experiments to obtain estimates of variance in each assay and 80% power to identify a two-fold difference in means with p<0.05.
Data exclusions	No data was excluded from analysis.
Replication	At least 3 independent experiments were performed for each dataset. Reproducibility confirmed the final conclusions and statistical analysis supported hypotheses reported. All attemps at replication were successful and no results were excluded.
Randomization	Experimental groups are identical except for the specific variable being tested and therefore randomisation is not required.
Blinding	Blinding was not required because all outcomes are measured objectively by automated machines

Reporting for specific materials, systems and methods

We require information from authors about some types of materials, experimental systems and methods used in many studies. Here, indicate whether each material, system or method listed is relevant to your study. If you are not sure if a list item applies to your research, read the appropriate section before selecting a response.

Materials & experimental systems

n/a	Involved in the study	
[ ]	[x]	Antibodies
[ ]	[x]	Eukaryotic cell lines
[x]	[ ]	Palaeontology and archaeology
[x]	[ ]	Animals and other organisms
[ ]	[x]	Human research participants
[x]	[ ]	Clinical data
[x]	[ ]	Dual use research of concern

Methods

n/a	Involved in the study	
[x]	[ ]	ChIP-seq
[x]	[ ]	Flow cytometry
[x]	[ ]	MRI-based neuroimaging

Antibodies

Antibodies used

goat anti-MAVS Ab (Cell signaling #3993) (1:1000 dilution)
goat anti- tubulin Ab (Abcam, #ab6046) (1:20,000 dilution)
RDye® 800CW Goat anti-Rabbit IgG (H + L), #926-32211 (1:20,000)
anti-IFN-α/β receptor (PBL Interferon Source) 1µg/ml
Control IgG2A (R+D Systems) 1µg/ml
goat anti-mouse immunoglobulin (Ig) antibody conjugated to β-galactosidase, Southern Biotechnology Associates, #926-32210, 1:15000
CA-specific antibodies (EVA365 and EVA366 National Institute of Biological Standards AIDS Reagents 1/50

Validation	Abcam datasheet provides evidence for use of anti-tubulin Ab by WB and cites 955 examples of published use file:///Users/newuser/Downloads/datasheet_6046.pdf<br>Cell signaling website contains evidence for MAVS detection by WB and cites 72 examples of published use https://www.cellsignal.com/products/primary-antibodies/mavs-antibody/3993<br>Suppression of IFN signaling using anti-IFN-α/β receptor and Control IgG2A was validated in PMID24196705 and 32852081<br>Detection of HIV infected cells by Capsid staining using anti-Capsid EVA365 and EVA366 and goat anti-mouse immunoglobulin (Ig) antibody conjugated to β-galactosidase was validated in PMID24196705, 19741482 and 19109163.

Eukaryotic cell lines

Policy information about cell lines

Cell line source(s)	ThP1 cells were purchased from Invivogen. HEK293T, U87, and Ghost cells were purchased from ATCC.
Authentication	All cell lines were originally purchased from ATCC or Invivogen. Both companies apply rigorous standards for cell line authentication using short-tandem repeat profiling. This confirms the identify of cells and detects misidentified, cross-contaminated, or genetically drifted cells.
Mycoplasma contamination	All cell lines were tested negative for mycoplasma infection.
Commonly misidentified lines (See ICLAC register)	No commonly misidentified cell lines were used in this study.

Human research participants

Policy information about studies involving human research participants

Population characteristics	We used healthy volunteers with no medical history or history of medication with age range from 21-50 and equal sex distribution
Recruitment	Recruitment was through advertising within the University, participants self selected but had to be self declared as healthy
Ethics oversight	UCL Research Ethics Committee

Note that full information on the approval of the study protocol must also be provided in the manuscript.
