# Supplementary material for: Evasion of cGAS and TRIM5 defines pandemic HIV
Source: Nat Microbiol. 2022 Oct 26;7(11):1762–76. doi: 10.1038/s41564-022-01247-0 (PMC9613477; doi:10.1038/s41564-022-01247-0)
Supplement: Supplementary file 3 — Structure data collection and refinement statistics. [file 41564_2022_1247_MOESM3_ESM.doc]

**Supplementary Table 2.** **Data collection and refinement statistics**

|  | HIV-1 (O) | SIVmac | SIVcpz | HIV-1 (M) Q50Y | HIV-1 (M) R120 | HIV1-1 (M) Q50Y/R120 |
| --- | --- | --- | --- | --- | --- | --- |
| **Data collection** |  |  |  |  |  |  |
| Space group | P2_1_ | P6 | P6_3_ | C2 | P6 | C2 |
| Cell dimensions   |  |  |  |  |  |  |
| *a*, *b*, *c* (Å) | 87.20, 151.73, 120.42 | 153.10, 153.10, 62.54 | 90.669, 90.669, 116.785 | 90.29, 156.61, 118.89 | 92.36, 92.36, 57.44 | 90.92, 157.31, 120.401 |
|     α, β, γ  (°) | 90.00, 105.03, 90.00 | 90.00, 90.00, 120.00 | 90.00, 90.00, 120.00 | 90.00, 107.33, 90.00 | 90.00, 90.00, 120.00 | 90.00, 107.28, 90.00 |
| Resolution (Å) | 75.86 - 3.00  (3.08 - 3.00)* | 29.29 – 2.25  (2.32 – 2.25)* | 78.52 – 2.05  (2.11 – 2.05)* | 78.30 – 3.15  (3.37 – 3.15)* | 46.66 – 2.30  (2.44 – 2.30)* | 76.01 – 3.29  (3.52 – 3.29)* |
| *R*_meas_ | 0.193 (1.369) | 0.180 (0.805) | 0.084 (1.018**)** | 0.247 (0.682) | 0.071 (1.509) | 0.229 (0.710) |
| *CC(1/2)* | 0.908 (0.282) | 0.994 (0.782) | 0.987 (0.857) | 0.943 (0.643) | 0.997 (0.503) | 0.976 (0.813) |
| <*I* / σ*I*> | 5.9 (1.8) | 9.7 (2.6) | 12.8 (2.3) | 3.6 (1.9) | 10.76 (1.00) | 5.0 (2.0) |
| Completeness (%) | 97.7 (97.5) | 99.4 (95.6) | 100.0 (100.0) | 95.4 (94.2) | 98.0 (97.0) | 99.7 (99.0) |
| Redundancy | 5.0 (4.7) | 5.2 (5.2) | 9.4 (9.5) | 2.5 (2.4) | 3.9 (4.0) | 3.3 (3.2) |
|  |  |  |  |  |  |  |
| **Refinement** |  |  |  |  |  |  |
| Resolution (Å) | 75.86 – 3.00 | 29.29 – 2.25 | 65.25 – 2.05 | 78.30 – 3.15 | 46.66 – 2.30 | 64.92 – 3.30 |
| No. reflections | 58577 | 37920 | 32525 | 25894 | 11698 | 24271 |
| *R*_work_ / *R*_free_ | 0.2144 / 0.2586 | 0.1889 / 0.2283 | 0.2018 / 0.2306 | 0.2046 / 0.2390 | 0.2423/ 0.2775 | 0.2390/0.2748 |
| No. atoms |  |  |  |  |  |  |
| Protein | 18753 | 4987 | 3354 | 9915 | 1748 | 9071 |
| Ligand/ion | - | - |  | - | 11 | - |
| Water | - | 513 | 115 | - | 14 | - |
| *B*-factors |  |  |  |  |  |  |
| Protein | 95.5 | 25.8 | 49.2 | 37.7 | 93.3 | 56.2 |
| Ligand/ion | - | - | - | - | 68.8 | - |
| Water | - | 33.8 | 44.7 | - | 66.8 | - |
| R.m.s. deviations |  |  |  |  |  |  |
| Bond lengths (Å) | 0.003 | 0.005 | 0.009 | 0.003 | 0.009 | 0.008 |
| Bond angles (°) | 0.606 | 0.906 | 1.491 | 0.527 | 2.017 | 1.479 |

*Values in parentheses are for highest-resolution shell.
