# Supplementary figures and images for: Evasion of cGAS and TRIM5 defines pandemic HIV
Source: Nat Microbiol. 2022 Oct 26;7(11):1762–76. doi: 10.1038/s41564-022-01247-0 (PMC9613477; doi:10.1038/s41564-022-01247-0)

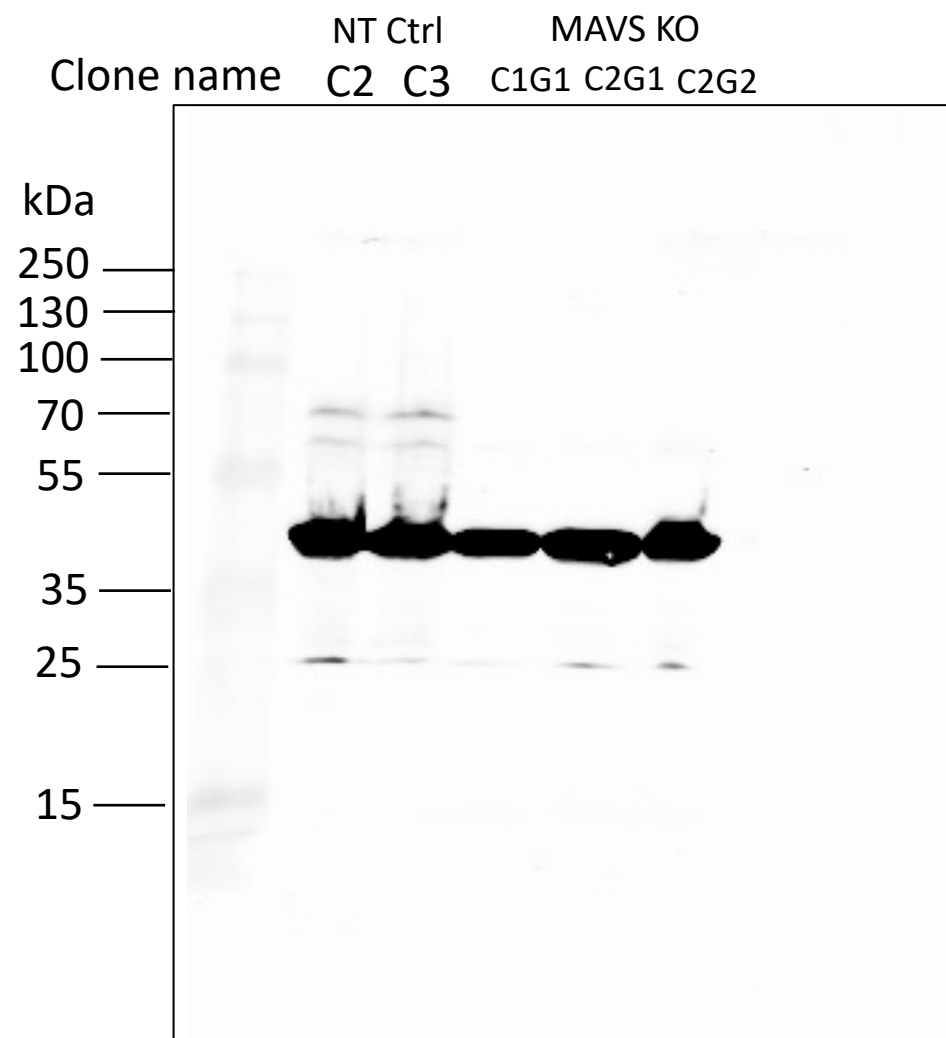

Supplement: Source Data Extended Data Fig. 2 — Raw MAVS western blot. [file 41564_2022_1247_MOESM12_ESM.pdf]
